# Exercise training promotes nerve cell repair and regeneration after spinal cord injury

**DOI:** 10.4103/NRR.NRR-D-24-01677

**Published:** 2025-06-19

**Authors:** Tianyu Zhai, Shuting Ren, Shenghao Qian, Caizhen Shi, Bingbing Wang, Can Zhang, Li Dan, Juan Shen, Feng Gao, Yanling Yang, Youlei Li, Lin Zhao

**Affiliations:** Yan’an Medical College of Yan’an University, Yan’an, Shaanxi Province, China

**Keywords:** astrocytes, axons, edema, exercise, inflammation, microglia, nerve regeneration, neurons, oxidative stress, spinal cord injury

## Abstract

Spinal cord injury is a severe neurological condition characterized by the permanent loss of nerve cell function and a failure in neural circuit reconstruction—key factors contributing to disability. Therefore, exploring effective strategies to promote the repair and regeneration of nerve cells after spinal cord injury is crucial for optimizing patient prognosis. The purpose of this paper is to conduct an in-depth review of the pathological changes in nerve cells after spinal cord injury and to present the state of research on the role of exercise training in promoting the repair and regeneration of nerve cells after spinal cord injury. In terms of the intrinsic growth capacity of neurons, disruptions in the dynamic balance between growth cones and the cytoskeleton, the dysregulation of transcription factors, abnormal protein signaling transduction, and altered epigenetic modifications collectively hinder axonal regeneration. Additionally, the microenvironment of neurons undergoes a series of complex changes, initially manifesting as edema, which may be exacerbated by spinal cord ischemia-reperfusion injury, further increasing the extent of nerve cell damage. The abnormal proliferation of astrocytes leads to the formation of glial scars, creating a physical barrier to nerve regeneration. The inflammatory response triggered by the excessive activation of microglia negatively impacts the process of nerve repair. Non-invasive interventions involving exercise training have shown significant potential in promoting nerve repair as part of a comprehensive treatment strategy for spinal cord injury. Specifically, exercise training can reshape the growth cone and cytoskeletal structures of neurons, regulate transcription factor activity, modulate protein signaling pathways, and influence epigenetic modifications, thereby activating the intrinsic repair mechanisms of neurons. Moreover, exercise training can regulate the activation state of astrocytes, optimize the inflammatory response and metabolic processes, promote astrocyte polarization, enhance angiogenesis, reduce glial scar formation, and modulate the expression levels of nerve growth factors. It also effectively helps regulate microglial activation, promotes axonal regeneration, and improves phagocytic function, thereby optimizing the microenvironment for nerve repair. In terms of clinical translation, we summarize the preliminary results of new drug research and development efforts, the development of innovative devices, and the use of exercise training in promoting clinical advancements in nerve repair following spinal cord injury, while considering their limitations and future application prospects. In summary, this review systematically analyzes findings relating to the pathological changes occurring in nerve cells after spinal cord injury and emphasizes the critical role of exercise training in facilitating the repair and regeneration of nerve cells. This work is expected to provide new ideas and methods for the rehabilitation of patients with spinal cord injury.

## Introduction

### Overview of nerve cells

In the complex and delicate network of the central nervous system (CNS), nerve cells are essential components primarily divided into neurons and glial cells (Liu et al., 2023b; Franco et al., 2025; Gao et al., 2025). Neurons receive external information through specialized dendrites and transmit electrical and chemical signals via their axons, enabling the rapid communication and integration of information. Neurons support the basic functionality of the nervous system and are key to the coordinated control of body behaviors, sensations, and responses (Hanslik et al., 2021; Ekelund et al., 2024).

Glial cells can be further subdivided into microglia, astrocytes, oligodendrocytes, and ependymal cells (Xu et al., 2023c; Lopez-Ortiz and Eyo, 2024). Microglia, which originate from yolk-sac-derived primitive macrophages of the mesoderm, play critical roles in CNS development, including synaptic pruning, the clearance of metabolic waste and pathogens, and the modulation of neuroinflammatory responses. They also support oligodendrocyte function and indirectly contribute to processes such as myelination (Isik et al., 2023; Beiter et al., 2024).

Astrocytes, derived from neuroectodermal progenitors, regulate synaptogenesis, provide metabolic support to neurons, and maintain the integrity of the blood–brain barrier. While they participate in immune regulation by releasing signaling molecules, their primary defensive roles complement the phagocytic activity of microglia (Jung et al., 2023; Schurhoff and Toborek, 2023). Oligodendrocytes are characterized by their multilayered membrane structure, which forms the myelin sheaths that wrap around axons. The myelin sheath is not only complex in structure but also contains cytoplasmic channels that connect to axons. During maturation, the characteristics of the oligodendrocyte cell membrane change to facilitate myelin formation. Functionally, oligodendrocytes accelerate nerve impulse conduction, maintain axonal integrity through metabolic support, and exhibit immunomodulatory effects.

Abnormalities in oligodendrocytes can lead to axonal degeneration (Kuhn et al., 2019; Simons et al., 2024). Ependymal cells, classified as neuroepithelial multiciliated cells, line the surfaces of the spinal cord and ventricles and can be categorized into various subtypes based on their distinct characteristics. These cells establish a blood-cerebrospinal fluid barrier through the formation of tight junctions, while their coordinated ciliary beating maintains cerebrospinal fluid homeostasis, enhances toxin clearance, and supports the transport of signaling molecules. In disease conditions, the morphology and function of ependymal cells change, allowing them to participate in the disease process. For example, after spinal cord injury (SCI), ependymal cells can proliferate, differentiate, and migrate to the injury site to facilitate repair (Deng et al., 2023; Xie and Li, 2024). In summary, neurons and glial cells each have unique characteristics in the CNS and work together to maintain the health and functionality of the system.

### Overview of spinal cord injury

As a major disease that poses a serious threat to the health of the nervous system, SCI is a significant global burden (Hersh et al., 2024). Each year, many new patients are added to the SCI population, with approximately 90% of cases resulting from traumatic events such as traffic accidents, falls, sports injuries, and violence (Baroudi et al., 2024). SCI involves damage to the structure and function of the spinal cord due to trauma or disease, resulting in paralysis that affects the sensory, motor, and autonomic nervous systems below the level of the injury (He et al., 2024a, b). SCI can be categorized based on the mechanism of injury into two primary types: traumatic and non-traumatic. Traumatic SCI is caused by an external physical impact, whereas non-traumatic SCI results from diseases or conditions such as infections, tumors, or degenerative disorders. Additionally, from the perspective of injury severity and anatomy, SCI can be further classified into complete and incomplete injuries. A complete injury entails a total loss of sensory and motor function below the injury level, while an incomplete injury retains some degree of neurological function (Schreiner et al., 2024). It is important to note that there are significant differences in the pathological manifestations and treatment strategies between these types of SCI (Baroudi et al., 2024; Yari et al., 2024).

### Relationship between spinal cord injury and nerve cells

**Figure 1:**
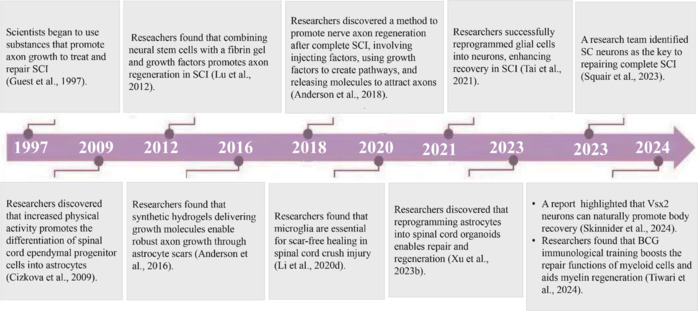
Important milestones in the research progress on the relationship between spinal cord injury and nerve cells. BCG: Bacillus Calmette–Guérin; SC: SC^Vsx2::Hoxa7::Zfhx3→lumbar^; SCI: spinal cord injury.

There is a close relationship between SCI and nerve cells. At the moment of injury, the primary damage is caused by acute mechanical trauma, which directly leads to the rupture of blood vessels, the collapse of the blood–spinal cord barrier (BSCB) (Jiang et al., 2023), the death of neurons and glial cells, the rupture of spinal nerve fiber bundles, and disruptions to the normal structure of the nervous system (Han et al., 2024b). Following this initial trauma, a series of complex secondary injuries occur, encompassing a variety of pathological processes such as inflammatory responses, ischemia-reperfusion injury, free radical injury, demyelination, astrocyte proliferation, and extracellular matrix remodeling (Fischer et al., 2024; Zavvarian et al., 2024). These intricate pathological processes severely impair nerve regeneration and synaptic plasticity, posing significant challenges to the recovery of SCI patients. An important milestone was reached in research into SCI when the relationship between SCI and nerve cells was unraveled (**Figure 1**).

Since the 1990s, scientists have been dedicated to exploring ways to promote axon growth and deactivate growth-inhibiting molecules and to lay a theoretical foundation for the effective treatment of nerve trauma (Bregman et al., 1995; Guest et al., 1997). In the 21^st^ century, research highlighted the importance of interactions between nerve cells and their microenvironment. For instance, studies have shown that physical activity influences spinal cord progenitor cells (Teng et al., 2006; Cizkova et al., 2009). Neural stem cells, when combined with a fibrin matrix and growth factors to form a gel, have been applied to repair completely transected spinal cords in rats. These stem cells can differentiate into various cell types, including neurons that can grow, extend axons, and form functional relays at the site of injury, significantly promoting axon growth and functional recovery (Lu et al., 2012). This suggests that a specific microenvironment can stimulate nerve regeneration after CNS injury.

It has been traditionally believed that astrocyte scars hinder axon regeneration (Bhatt et al., 2024; Li et al., 2024a); however, new experimental evidence challenges this view. Experiments have demonstrated that merely preventing or eliminating scars does not facilitate spontaneous axon regeneration. On the contrary, a continuous supply of specific growth factors is crucial for promoting axonal regeneration. The formation of scars may actually play a supportive role in this process. RNA sequencing has further revealed that cells within scars express numerous molecules that support axon growth, reinforcing the emerging perspective that scars contribute positively to axon regeneration (Anderson et al., 2016).

With the development of regenerative medicine, the plasticity of nerve cells and its key role in injury repair have been further confirmed (Anderson et al., 2018). Researchers have successfully reprogrammed glial cells into neurons and promoted recovery after SCI (Tai et al., 2021). Furthermore, a study found that spinal cord compression injury can achieve scar-free healing; this relies on functional microglia to promote axon regeneration, providing a new target for the treatment of SCI (Li et al., 2020a). Human astrocytes can be directly reprogrammed into early neuroectodermal cells to induce the formation of spinal cord-like organs. After transplantation into a mouse model of SCI, human astrocytes showed amazing survival and differentiation ability, and established synaptic connections with host neurons, opening up a new avenue in the field of cell replacement therapy (Xu et al., 2023b). More excitingly, a research team recently found that spinal cord (SC)^Vsx2::Hoxa7::Zfhx3→lumbar^ neurons are crucial in repairing complete SCI (Squair et al., 2023). The regeneration of SC neurons and their reconnection with neurons in the projected target area have potent significance in the recovery of motor function and provide a clear cellular target for the treatment of SCI, which has far-reaching clinical implications. In 2024, Skinnider et al. have made new breakthroughs in the study of the cellular and molecular dynamical characteristics of human paralysis, and identified Vsx2 neurons with recovery ability. In addition, lateral hypothalamic (LH) Vglut2 neurons in the LH area of the brain play a key role in the recovery of walking function after SCI. The activation of these neurons not only optimizes gait performance but also enhances the plasticity of the nervous system by establishing functional connections with glutamatergic neurons in the ventral gigantocellular nucleus. At the spinal cord level, even if injury results in some damage to nerve projections, some projections remain intact, and exercise interventions can effectively promote the reorganization of these residual projections. For example, in a rat model, robot-assisted rehabilitation combined with deep brain stimulation of the lateral hypothalamic area significantly increased the number of projections arising from ventral gigantocellular nucleus neurons and reaching the lumbar spinal cord, thereby effectively improving walking function (Cho et al., 2024). Another study found that Bacillus Calmette–Guérin immune training enhanced the repair function of myeloid cells and improved myelin regeneration, during which the phagocytic activity of microglia increased significantly (Tiwari et al., 2024). These results not only confirm the importance of interactions between nerve cells in the repair of SCI but also provide a scientific basis for the development of new therapeutic strategies (Tiwari et al., 2024; Timofeeva et al., 2025).

Although there exists a large amount of evidence that exercise contributes to the repair of neurological function after SCI (Gaspar et al., 2019; Pelletier, 2023), how exercise training promotes improvements in neurological function, especially the molecular mechanisms involved in its promotion of nerve cell repair and regeneration, remain largely unresolved. This review provides a comprehensive analysis of the pathological evolution of nerve cells after SCI. Research has clearly highlighted the positive role of exercise training in promoting nerve regeneration and proposed effective intervention strategies. In terms of therapeutic innovations, the review examines the pathological changes from multiple dimensions and focuses on the specific effects of exercise training on different types of nerve cells. The core objective of this article is to clarify the internal mechanisms by which exercise training promotes nerve repair with the aim of constructing a novel theoretical framework for the field of SCI rehabilitation. This aligns with cutting-edge trends in nerve regeneration research and aims to facilitate the clinical translation of research findings and ultimately significantly improve the rehabilitation prognosis of patients.

## Literature Retrieval Strategy

The references covered in this review are mainly from literature published from 1990 to 2024, of which more than 50% of articles are from 2021 to 2024. The literature search was based on the PubMed database, the US Food and Drug Administration (FDA), the North American Clinical Trial Registry, the Chinese Clinical Trial Registry, and the National Institutes of Health (NIH). The number of literature items included was approximately 300. For the precise retrieval strategy, “spinal cord injury,” “exercise training,” “growth cone and cytoskeleton,” “transcription factor,” “protein signal transduction molecule,” “epigenetics,” “microglia,” “astrocytes,” “microenvironment,” “inflammation,” and “functional phenotype” were used as core keywords, and multiple combinations of these were used in searches. Once the literature search was completed, studies that were unrelated to the topic or repetitive were excluded based on strict screening criteria. Additionally, literature and materials with poor correlation were removed to ensure the academic rigor of the review.

## Neuronal Repair and Regeneration After Spinal Cord Injury

### Changes of intrinsic growth ability of neurons after spinal cord injury

**Figure 2:**
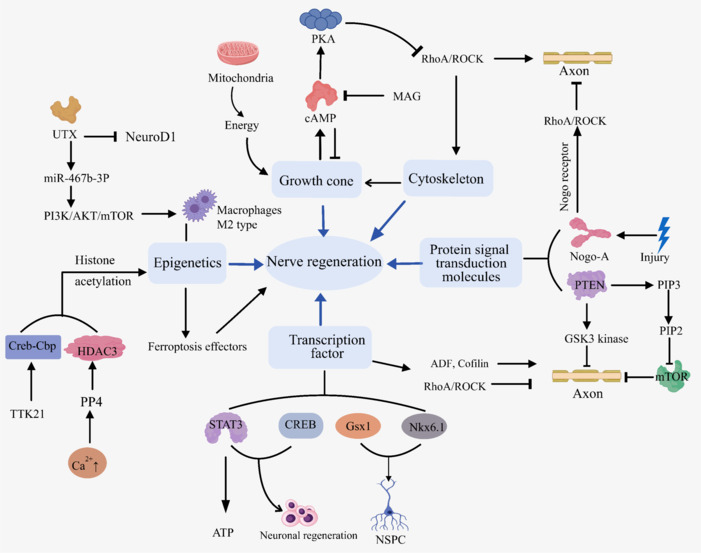
A mechanism diagram of changes in intrinsic neuronal growth capacity after SCI. Created with MedPeer (medpeer.cn). This figure illustrates the mechanisms of action in nerve regeneration, including growth cones, cytoskeleton, transcription factors, protein signaling molecules, and epigenetics. ADF: Actin-depolymerizing factor; AKT: protein kinase B; ATP: adenosine triphosphate; cAMP: cyclic adenosine monophosphate; CREB: cAMP response element-binding protein; Creb-Cbp: CREB-binding protein; GSK3: glycogen synthase kinase 3; Gsx1: GS homeobox 1; HDAC3: histone deacetylase 3; MAG: myelin-associated glycoprotein; mTOR: mechanistic target of rapamycin; NeuroD1: neuronal differentiation 1; Nkx6.1: NK6 homeobox 1; NSPC: neural stem/progenitor cell; PI3K: phosphoinositide 3-kinase; PIP2: phosphatidylinositol 4,5-bisphosphate; PIP3: phosphatidylinositol (3,4,5)-trisphosphate; PKA: protein kinase A; PP4: protein phosphatase 4; RhoA: ras homolog family member A; ROCK: rho-associated coiled-coil containing protein kinase; SCI: spinal cord injury; STAT3: signal transducer and activator of transcription 3; UTX: ubiquitously transcribed tetratricopeptide repeat, X chromosome.

After SCI, axons are often damaged or broken, resulting in a serious loss of neurological function. Therefore, stimulating the intrinsic growth potential of neurons to promote axonal regeneration is particularly important in the treatment of nerve repair after SCI (Hutsonand Di Giovanni, 2019). The intrinsic growth mechanisms of neurons involve the formation of growth cones and the dynamic regulation of the cytoskeleton, the transduction of transcription factors and protein signaling molecules, and fine regulation at the epigenetic level. We provide a mechanism diagram of the changes in intrinsic neuronal growth capacity that occur after SCI in **Figure 2**.

#### Growth cone and cytoskeleton

A growth cone is a specialized cytoskeletal structure at the end of an axon. Its highly dynamic nature is reflected in the continuous advancement of internal microtubule bundles and the formation of contact points with anterograde actin bundles (Tran et al., 2018). These contact points are expanded by *in vivo* protein complexes that drive the expansion of growth cones and axon regeneration (Zhang et al., 2015; Domínguez-Romero and Slater, 2021). However, this fine regulatory mechanism can be seriously damaged after SCI. In this process, the growth cone swells to form a spherical structure and continues to retract, eventually causing axonal degeneration (Stewart et al., 2024). This change not only reflects the instability of the structure of the growth cone itself but also reveals the disorder in its internal control mechanism. Moreover, the distribution of microtubules in the spinal cord becomes disordered, and the number of microtubules gradually decreases until they disappear, which further aggravates the damage in neurons (Blanquie and Bradke, 2018).

As an important part of the cytoskeleton, microtubule stability is essential for maintaining physiological functions such as neuronal morphology, axonal transport, and neurotransmitter release. In addition, the process of cytoskeleton reorganization after nerve injury, especially the formation of the growth cone, is highly dependent on the energy supply from mitochondria (Petrova et al., 2021). An increase in the number of mitochondria and improvements in their motor ability play significant roles in promoting the formation of growth cones and optimizing axon regeneration (Liet al., 2018; Han et al., 2020).

#### Transcription factors

In the process of SCI, transcription factors accurately regulate the transcriptional activity of genes by binding to specific regions of DNA, thereby guiding the survival, regeneration, and functional recovery of neurons (Shi et al., 2024). Signal transducer and activator of transcription 3 (STAT3) is a core transcription factor of axon regeneration. Its activation can not only promote the survival of neurons but also regulate the expression of regeneration-related genes. In mitochondria especially, the expression of STAT3 plays a key role in regulating the intracellular ATP level and supporting axon growth after SCI (Luo et al., 2016). The positive role of cAMP-response element binding protein (CREB) in synaptic plasticity and long-term memory formation further highlights the importance of transcription factors in nerve regeneration (Huang et al., 2020). In addition to STAT3 and CREB, the lentivirus-mediated expression of genomic screened homeobox 1 (Gsx1) and NK6 homeobox 1 (Nkx6.1) can activate neural stem/progenitor cells. These transcription factors can also reduce astrocyte proliferation and glial scar formation in the lesion site, providing a more favorable microenvironment for nerve regeneration.

The RhoA/ROCK signaling pathway plays a central role in regulating axonal regeneration. RhoA, a GTPase, inhibits actin depolymerization by binding to ROCK, thereby impeding growth cone dynamics and axonal regrowth after SCI (Cherfils and Zeghouf, 2013). Conversely, actin-depolymerizing factors, such as ADF and cofilin-1, promote microtubule protrusion and axon elongation by degrading dense actin structures, and moreover, this process is crucial for neural development (Tedeschi et al., 2019). RhoA exhibits a dual role depending on cellular context (Stern et al., 2021). While its knockdown in neurons enhances neurite growth, its inactivation in astrocytes abolishes regenerative effects and exacerbates motor dysfunction, underscoring its cell-specific regulatory complexity (Govek et al., 2005; Tan et al., 2011; MacKay and Kumar, 2014). This highlights the need to explore interactions among transcription factors, signaling molecules (e.g., *RhoA*, *Cdc42*, *Rac1*), and their regulatory networks in SCI recovery (Ruchhoeft et al., 1999; Roy et al., 2021). Cyclic adenosine monophosphate (cAMP) further modulates the RhoA/ROCK pathway. Elevated cAMP levels activate protein kinase A, which suppresses RhoA-ROCK signaling to facilitate axon regeneration (Zhou et al., 2022). However, inhibitory factors such as myelin-associated glycoprotein counteract this effect by reducing the cAMP concentration, leading to growth cone collapse and axonal retraction (Meyer-Franke et al., 1998; Aglah et al., 2008). The interplay between these opposing signals underscores the challenges faced in achieving functional nerve repair post-SCI.

#### Protein signaling transduction molecules

Intracellular protein signal transduction molecules are components of a sophisticated and complex signal transduction network (Xu et al., 2021) that plays a vital role in the cell response to external stimuli. This network involves the fine regulation and interaction of various molecules, such as enzymes, transcription factors, receptors, and kinases. Dynamic changes in the network are closely related to the pathophysiological process of SCI, especially through their effect on related inflammatory responses, apoptosis, and axonal regeneration inhibition (Park et al., 2024).

After SCI, the expression of myelin-related inhibitors, such as Nogo-A, is significantly upregulated. These activate the downstream RhoA/ROCK signaling pathway by binding to Nogo receptors on the surface of neurons, leading to the depolymerization of neuronal cytoskeleton proteins and inhibiting axon growth (Dubreuil et al., 2003). Moreover, phosphatase and tensin homolog (PTEN), an important intrinsic factor of nerve growth failure after SCI (Kawano et al., 2012), generates phosphatidylinositol 4,5-bisphosphate by dephosphorylating phosphatidylinositol 3,4,5-trisphosphate. In turn, the former inhibits the activation of mammalian target of rapamycin (mTOR) signaling, blocks protein translation and cell growth, and poses a serious challenge to axon regeneration. In addition, the activation of PTEN triggers the activation of GSK3 kinase and exacerbates the collapse of fibrous actin units and the retraction of growth cones (Maehama and Dixon, 1998; Dontchev and Letourneau, 2002).

Under normal conditions, the growth of axons depends on the extension of actin filament pseudopods and plate pseudopods. These processes are actively regulated by attractive cues, such as netrin-1, neurotrophic factors, and laminin, during development (Bennison et al., 2020). Therefore, an in-depth understanding of the mechanisms involving signal transduction molecules after SCI is crucial for the development of effective treatment strategies.

#### Epigenetic

Epigenetic regulation, a complex and delicate biological process, can affect the functional characteristics and behavior patterns of cells by regulating gene expression patterns without changing DNA sequences (Tomé and Almeida, 2024). Its core mechanism widely involves the regulation of DNA methylation, multiple forms of histone modifications (e.g., acetylation, methylation, phosphorylation, and ubiquitination), and non-coding RNAs (e.g., microRNAs and long non-coding RNAs) (Gupta et al., 2023). In the context of nervous system injury, especially SCI, dynamic changes in epigenetic modifications are involved in key processes of axon growth (Liu et al., 2024b).

Experimental evidence from a SCI model revealed that the intraperitoneal injection of the small molecule activator TTK21 (which mainly acts on CBP/p300 histone acetyltransferases and regulates gene expression by enhancing their activity) can effectively activate cyclic adenosine monophosphate response element binding protein, enhance its acetyltransferase activity, mediate histone acetylation, and induce the germination and regeneration of spinal cord axons (Hutsonand Di Giovanni, 2019). In addition, the increase in calcium ion concentrations in the body caused by peripheral nerve injury can activate protein phosphatase 4, lead to the dephosphorylation of histone deacetylase 3 (Qi et al., 2018), enhance the level of histone acetylation, and promote the growth of the dorsal root ganglion (Hervera et al., 2019). A study suggested that epigenetic regulation has significant potential in promoting nerve regeneration. Ferroptosis, as an iron-dependent cell death, is mainly characterized by the accumulation of lipid peroxidation and abnormal iron metabolism. Epigenetic regulation also affects ferroptosis effectors in cells through transcription and translation, regulates the expression of iron metabolism-related genes, reduces ferroptosis after SCI, and reduces nerve damage (Lan et al., 2023).

Lysine demethylase 6A (Kdm6a, also known as UTX) is also involved in epigenetic regulation after SCI (Liu et al., 2024a). UTX can form an epigenetic complex with miR-24, negatively regulate the expression of neurogenic differentiation factor 1, and inhibit axonal regeneration (Roidland Hacker, 2014). Notably, UTX can also upregulate the expression of miR-467b-3p, induce interactions between endothelial cells and macrophages, activate the PI3K/AKT/mTOR signaling pathway, and promote the polarization of macrophages into M2 subtypes (**Figure 2**). Therefore, UTX promotes angiogenesis and neurological functional recovery after SCI at the epigenetic level (Peng et al., 2023).

### Changes in neuronal growth microenvironment after spinal cord injury

The microenvironment of neuron growth after SCI undergoes pathological changes, such as intramedullary hemorrhage, oxidative stress, inflammatory cell infiltration, and glial scar formation. These may lead to spinal cord edema and spinal cord ischemia-reperfusion, as well as disruptions in spinal cord axon structure and function (Tator and Fehlings, 1991).

#### Edema

After SCI, water rapidly accumulates in the damaged area, leading to the formation of edema. This process is particularly prominent during the acute phase of secondary injury and plays a critical role in the progression of SCI. The severity of edema, including its extent and location, is strongly correlated with clinical outcomes, especially in terms of motor recovery (Seblani et al., 2023). Edema typically begins within minutes after the initial injury, with a fluid-filled cavity evolving within 48 hours and potentially persisting for up to 14 days post-injury (Tator and Fehlings, 1991).

The development of edema involves two main mechanisms: cytotoxic edema and vasogenic edema. Cytotoxic edema arises from the swelling of neurons and astrocytes due to cellular dysfunction. Astrocytes, which are highly sensitive to changes in potassium (K^+^) levels, are particularly susceptible. The process is triggered by factors such as local inflammatory mediators, ATP depletion, and the release of arachidonic acid. These events impair the function of the ATP-dependent Na^+^-K^+^-ATPase pump, leading to an ion imbalance and influx of water into cells, causing them to swell (Faden et al., 1987). Elevated potassium levels further exacerbate astrocyte swelling and lower the extracellular pH, worsening the condition. Additionally, cytotoxic edema disrupts the ion concentration gradient, promoting water movement across the blood–brain barrier and into the spinal cord parenchyma, initiating the first stage of CNS tissue swelling. Vasogenic edema occurs due to the breakdown of the BSCB, allowing fluid and proteins to leak into spinal cord tissue. The permeability imbalance in the blood–CNS barrier further aggravates edema, forming permeable pores and contributing to tissue damage (Lo et al., 1987; Kimelberg, 2004; Rungta et al., 2015). Edema not only worsens the primary injury but also increases the intrathecal pressure, reducing blood flow to the spinal cord. This can lead to additional complications, such as bleeding, further destruction of the BSCB, and neuronal death (Zhang et al., 2019). Understanding these mechanisms is crucial for developing targeted therapies to mitigate edema and improve outcomes for SCI patients.

#### Spinal cord ischemia–reperfusion injury

Spinal cord ischemia–reperfusion injury (SCII) is a complex pathological process accompanied by the development of edema after SCI and involving extensive interactions among multiple cells and molecules (Lee et al., 2024; Liu et al., 2024a). In this process, spinal cord tissue first undergoes hypoxia due to the limited blood supply. The subsequent reperfusion and reoxygenation not only fail to alleviate the damage but often lead to further tissue deterioration, accompanied by a severe inflammatory response. Inflammation plays a central role in SCII and is the main cause of delayed neuronal death (Zhu et al., 2013). Inflammatory cells such as microglia, lymphocytes, macrophages, astrocytes, and neutrophils are involved in the release of CXCL13, interleukins (IL-1, IL-6, IL-8, and IL-10), TNF-α, and other cytokines and chemokines that further aggravate the inflammatory response and injury of spinal cord tissue (Zhu et al., 2013; Xie et al., 2024). NF-κB signaling plays a key role in this process (Jia et al., 2019; Jin et al., 2021), and its activation regulates the expression of target genes and triggers a series of inflammatory reactions. The excessive inflammatory reactions may further aggravate spinal cord tissue injury (Jin et al., 2021; Wu et al., 2024).

Oxidative stress is another core mechanism of secondary ischemia–reperfusion injury. Spinal cord ischemia induces the massive production of reactive oxygen species (ROS) (Fassbender et al., 2011; Oruc et al., 2024). These ROS damage phospholipid membranes, leading to lipid peroxidation and increased vascular permeability and eventually to cell death and reperfusion injury (International, 2024). As the main source of ROS, mitochondria exhibit damaged structure and function after SCII, which further aggravates the production of ROS, affects the synthesis and release of neurotransmitters, leads to an imbalance in neurotransmitters, and aggravates dysfunction after SCII (Stadtman, 2006; Lin et al., 2021; Sun et al., 2023a, b).

**Figure 3:**
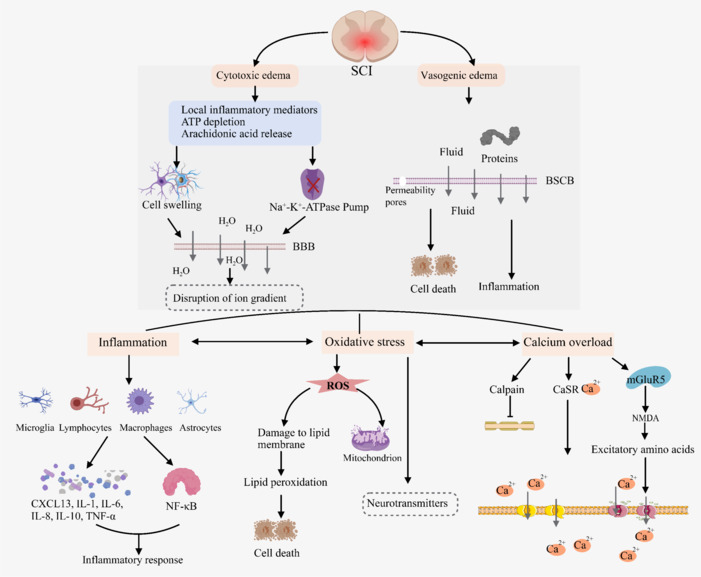
Mechanism diagram of changes in the neuronal growth microenvironment after SCI. Created with MedPeer (medpeer.cn). After SCI, a series of dynamic changes occur in the neuronal microenvironment. These changes include edema in the early stage of SCI and subsequent spinal cord ischemia‒reperfusion injury. Spinal cord ischemia‒reperfusion injury further exacerbates this adverse environment, triggering complex molecular changes in the inflammatory response, oxidative stress, calcium homeostasis disruption, and excitatory amino acid toxicity. BSCB: Blood‒spinal cord barrier; CaSR: calcium-sensing receptor; CXCL13: C-X-C motif chemokine ligand 13; IL-1: interleukin-1; IL-6: interleukin-6; IL-8: interleukin-8; IL-10: interleukin-10; mGluR5: metabotropic glutamate receptor 5; NF-κB: nuclear factor kappa-light-chain-enhancer of activated B cells; NMDA: N-methyl-D-aspartate; ROS: reactive oxygen species; SCI: spinal cord injury; TNF-α: tumor necrosis factor-alpha.

Calcium (Ca^2+^) is also a crucial factor in the pathophysiological processes of SCII (Bano and Ankarcrona, 2018). A variety of mechanisms promote Ca^2+^ flow into nerve cells and induce intracellular Ca^2+^ overload. Activating Ca^2+^-dependent calpain activity leads to cytoskeletal protein degradation, myelin protein vesicle changes, axonal degeneration, cell structure destruction, and ultimately neuronal cell death. Calcium-sensing receptor-mediated signal transduction also plays an important role in this process (Conigrave and Ward, 2013; Sun et al., 2017a), and calcium-sensing receptor activation may lead to intracellular calcium overload. Furthermore, large amounts of excitatory amino acids are released, which bind to N-methyl-D-aspartate receptors, open calcium channels, aggravate intracellular calcium overload, and cause secondary damage to spinal nerve cells (Sun et al., 2017b; Liu et al., 2020). Metabotropic glutamate receptor 5 plays a key role in regulating the excitatory toxicity of spinal neurons (Qian et al., 2021). For a better understanding, we provide a diagram of the mechanisms involved in neuronal growth microenvironment changes after SCI in **Figure 3**.

### Effects of exercise training on the intrinsic growth capacity of neurons

This section summarizes the effects of exercise training on various aspects after SCI, including promoting growth cone and cytoskeleton recovery, regulating transcription factors, affecting protein signal transduction, and regulating gene expression through epigenetics. These findings provide a crucial foundation for developing personalized rehabilitation strategies after SCI. For greater clarity, the effects of exercise training on the intrinsic growth capacity of neurons are summarized in **Additional Table 1**.

**Additional Table 1:** Effects of exercise training on the intrinsic growth capacity of neurons

Topic	Species	Form of motion	Regulatory mechanism	Result	Reference
Effects of exercise training on the growth spine and cytoskeleton	Rat	Treadmill training (15 min/time, 5 d/wk, 5 cm/s, gradually increased to 15 cm/s)	Upregulates NF200, Synl, PSD95, α-, β-, γ-tubulin, MAP2; regulates microtubule dynamics	Increases neuronal growth cone reactivity; promotes dendritic spine formation, synaptic transmission, and axon regeneration	Tang et al., 2024
	Rat	Swimming training (15 min/d, gradually increased to 60 min/d, 5 d/wk, for 4 wk)	Upregulates GAP-43	Promotes spinal nerve growth; improves hippocampal plasticity; alleviates depressive behavior	Kawasaki et al., 2018; Liu et al., 2018
	Rat	Aerobic or strength training	Adjusts cytoskeleton structure; regulates cytoskeleton-related proteins	Enhances cellular support; affects cytoskeleton dynamics	Oh et al., 2009; Kim et al., 2018; Kiss Bimbova et al., 2022
	Rat	Endurance training, treadmill training (8 m/min, 30 min/time, 1 time/d, 6 d/wk, for 6 wk)	Activates ERK1/2, PLCγ-PKC, PLC-CAMKII signaling pathways	Stimulates axonal regeneration	Oh et al., 2009; Kim et al., 2018; Kiss Bimbova et al., 2022
	Rat	Cycling training	Improves blood flow and nutrition supply in SCI area	Provides material basis for growth cone regeneration	Sandrow-Feinberg and Houlé, 2015
	Rat	Moderate intensity exercise (for 6 wk, week 6 exercise programme: 30 min/d, TI = 50 %, MRD/TS = 734, v = 48-50 m/min)	Upregulates GAP-43 and CAP-1	Promotes the recovery of neural plasticity	Rahmati and Kazemi, 2019
	Rat	Resistance training and combined endurance and resistnce training	Delays functional recovery; but promotes myelin fiber maturation	Promotes the maturation of myelin fibers	Ilha et al., 2008
Effects of exercise training on transcription factors	Rat	Treadmill training (initial speed 2 m/min, until 6 m/min, 15 min/time)	Promotes OPC proliferation, OL maturation, MBP expression, and myelin sheath thickness	Enhances motor function and nerve conduction efficiency	Gomez-Pinilla et al., 2012; Su et al., 2024
	Rat	Multisport training (treadmill, water treadmill, passive bicycling, wheeled running, and swimming)	Upregulates BDNF levels	Promotes neural plasticity	Bilchak et al., 2021
	Rat	Running wheels training	Upregulates BDNF, p-synapsin I, p-CREB, p-CaMKII and GAP-43	Improves axonal transport and CNS resistance to neurological decline	Gomez-Pinilla et al., 2012
	Rat	Sports training	Increases MBP, PGC1α, and antioxidant enzymes	Enhances OL resistance to ROS; promotes myelin repair	Hyatt et al., 2019
		BWSTT (20 min/time, 2 times/day, 5 d/wk, for 4 wk); water treadmill training (10-15 m/min, 5 min/wheel, 3 rounds, 5 min interval between wheels)	Upregulates BDNF/TrkB-CREB signaling pathway	Improves BSCB injury and functional recovery	Li et al., 2019, 2020a, b; Ying et al., 2020;
	Rat	Treadmill exercise (5 d/wk, for 2 wk, female mice were repeated at a speed of 20 m/min 4 times, lasting for 2 min, with a 5-min break between them. Male mice were repeated at a speed of 10 m/min for 1 h	It is closely related to BDNF regulation	Enhances motoneuron regeneration	Acosta et al., 2017
	Man	FES-LEC or ACE training	Affects the abundance of GLUT-4, AMPK and PGC1a	Close to trained and innervated muscles	Gorgey et al., 2017
Effects of exercise training on protein signal transduction	Rat	BWSTT (8 m/min, 1 time/d, 10 min/time, 6 d/wk)	Reduces TGF-β1 and HIF-1α; increases MAP1B, NSE, and VIM; inhibits Nogo-NgR pathway	Improves spasticity and motor function; promotes neuronal axon repair	Beverungen et al., 2020; Bilchak et al., 2021; Li et al., 2022a, c
	Rat	BWSTT (6 m/min, 20 min/time, 2 times/d, 5 d/wk, for 4 wk)	Induces BDNF-TrkB signaling; regulate Ras/ERK, PLC-γ1, GAD-65/67, KCC2	Optimizes GABAergic inhibitory neural circuits; reduces neuronal overexcitation	Li et al., 2020b, c
	Rat	Exercise training (e.g., constant current alternating current (60 Hz) electric shock for flexor exercise training)	Upregulates NT-3, NT-4, GDNF, and VEGF	Promotes neuronal survival and axon regeneration	Huang and Reichardt, 2001; Gómez-Pinilla et al., 2007; Keefe et al., 2017
	Cat	BWSTT	Promotes CPG and local neural circuit remodeling	Improves walking function	Barrière et al., 2008; Molinari, 2009; van Hedel and Dietz, 2010
	Zeba fish	Swimming training (60% of critical speed for training, 6 h/d, 5 d/wk)	V2a-IN promotes NSPC proliferation and neurogenesis	V2a-IN promotes NSPC proliferation and neurogenesis	Chang et al., 2021
Regulation of epigenetics by exercise training	Rat	Treadmill training	Upregulates BDNF; demethylates BDNF promoter; reduces CSPG expression	Enhances BDNF transcriptional activity; optimizes axon structure; promotes myelin formation	Ilha et al., 2011
	Rat	High-capacity resistance/endurance exercise for progressive weight-bearing wheel running	Increases histone acetylation; reduces methylation	Promotes nerve regeneration; change chromatin structure	Ghosh et al., 2022
	Rat	Swimming training (2 times/d, 5 d/wk, for 4 wk)	Upregulates miR-21; inhibits PDCD4	Protects against neuronal damage; provides epigenetic regulatory mechanism	Li et al., 2020a; Wan et al., 2023

ACE: Arm cycling ergometry; AMPK: adenosine monophosphate-activated protein kinase; BDNF: brain-derived neurotrophic factor; BWSTT: body-weight-supported treadmill training; CAP-1: cyclase-associated protein 1; cpg: central pattern generator; CREB: cAMP-response element binding protein; ERK1/2: extracellular signal-regulated kinase 1/2; FES-LEC: functional electrical stimulation of lower limb circulation; GAP-43: growth-associated protein 43; GLUT-4: glucose transporter type 4; HSP72: heat shock protein 72; MAP2: microtubule-associated protein 2; MBP: myelin basic protein; MIE: moderate-intensity exercise; MRD/TS: mean running distance/training segment; NF200: neurofilament 200; OPC: oligodendrocyte precursor cell; PGC1α: peroxisome proliferator-activated receptor gamma coactivator 1-alpha; PLCγ-PKC: phospholipase c gamma-protein kinase C; PSD95: postsynaptic density protein 95; Syn1: synapsin I; TI: training intensity

#### Effects of exercise training on growth cone and cytoskeleton

The mechanism by which exercise training promotes neurological recovery after SCI has been a focal topic of neuroscience research (Fu et al., 2016). A related study revealed that exercise training enhances the expression of neurofilament protein 200, synapsin I, and postsynaptic density protein 95, which are crucial for dendritic spine formation, synaptic transmission, and axonal regeneration (Tanget al., 2024). Furthermore, exercise training boosts the expression of several microtubule-associated proteins, including α-, β-, and γ-tubulin and microtubule-associated protein 2 (MAP2). These proteins enhance the responsiveness of neuronal growth cones by regulating microtubule dynamics, promoting the outward growth of neurites, and facilitating the intracellular transport of essential substances (Tanget al., 2024).

Notably, exercise training also stimulates neurite growth and axon regeneration by inhibiting microtubule de-tyrosination and accelerating growth cone kinetics. The S96 site of growth-associated protein 43 (GAP43) exhibits peak phosphorylation levels during the formation of highly active growth cones at the elongated ends of neurons (Kawasakiet al., 2018). GAP43 augments the axon length and migration capacity of cortical and spinal neurons, fosters spinal nerve growth after SCI, and is intimately linked to functional recovery (Martínez-Torres et al., 2024). A study has shown that swimming exercise can improve hippocampal plasticity and alleviate depressive behaviors by upregulating the expression of GAP43 (Liuet al., 2018).

In addition to its effects on microtubule dynamics, exercise training is able to modulate the dynamic balance within the cytoskeleton after SCI through multiple pathways. Aerobic exercise or strength training adjusts the structures of microfilaments and intermediate fibers in the cytoskeleton, causing them to form tighter bonds and thereby enhancing the robustness of the cellular support (Miotto et al., 2017; Villarreal-Salazar et al., 2022). Exercise training also modulates the expression of cytoskeleton-related proteins, which further affect the dynamics of the cytoskeleton. These changes include the activation of the extracellular signal-regulated kinase (ERK)1/2, phospholipase C (PLC) gamma–protein kinase C, and PLC–calmodulin-dependent protein kinase II signaling pathways, which phosphorylate downstream molecules such as c-Jun N-terminal kinase and thereby stimulate axonal regeneration (Kiss Bimbova et al., 2022; Kimet al., 2018; Ohet al., 2009).

Exercise training can substantially enhance blood flow and the nutrient supply in the SCI area, furnishing the tissue with essential materials for growth cone regeneration (Sandrow-Feinberget al., 2015). GAP-1 is a GTPase-activating protein that can affect cell growth and differentiation and is involved in cytoskeleton reorganization and cell migration. The expression levels of GAP-43 and GAP-1 are significantly influenced by exercise intensity. Moderate-intensity exercise is deemed most favorable for the expression of GAP-43 and CAP-1, which facilitate the restoration of neural plasticity (Rahmati and Kazemi, 2019). Diverse exercise modalities exert notable impacts on nerve regeneration. For instance, endurance training was reported to augment nerve regeneration in rats subjected to experimental trauma, whereas resistance training and combined endurance and resistance training postponed functional recovery but accelerated the maturation of myelin sheath fibers (Ilhaet al., 2008). In summary, as an efficacious rehabilitation approach, exercise training demonstrates extensive application potential in advancing the recovery of growth cones and cytoskeleton function post-SCI.

#### Effects of exercise training on transcription factors

A study has indicated that early intervention with treadmill training can considerably propel the recovery of motor function in adult rats following incomplete closed spinal cord compression injury (Li et al., 2024b). This recovery was primarily attributed to the promotion of oligodendrocyte precursor cell proliferation and oligodendrocyte maturation, along with the augmentation of myelin basic protein expression and myelin sheath thickness. These alterations collectively enhance motor function and nerve conduction efficiency in the hind limbs. Additionally, exercise has been shown to elevate the level of brain-derived neurotrophic factor (BDNF), thereby fostering nerve conduction and neural plasticity (Gomez-Pinilla et al., 2012; Su et al., 2024). This elevation in BDNF levels is not confined to TT; other forms of exercise, such as traditional TT, water TT, passive cycling, wheeled running, and swimming, can also substantially increase BDNF levels, suggesting that exercise’s regulation of BDNF is pervasive and extensive (Bilchak et al., 2021).

Shamsnia et al. (2025) found that exercise before SCI increased the level of BDNF and related molecules in the spinal cord and hippocampus of animals, which had a certain neuroprotective effect. However, SCI reduced BDNF levels and affected axonal transport. Fortunately, the level of hippocampal BDNF in SCI animals can be close to normal levels after exercise, and moreover, exercise can also effectively offset the decrease in hippocampal phospho-synapsin I caused by SCI. In addition, exercise can increase the levels of phospho-CREB, phosphorylated calmodulin-dependent protein kinase II, and GAP-43 in the hippocampus of animals, further emphasizing that an active lifestyle may enhance the ability of CNS to resist neurological and cognitive decline after SCI (Gomez-Pinilla et al., 2012). Exercise training not only increases the levels of myelin basic protein and peroxisome proliferator-activated receptor gamma coactivator 1 alpha but also the content of antioxidant enzymes. It may additionally enhance the ability of oligodendrocytes to resist ROS injury and promote myelin repair through the stimulation of mitochondrial biogenesis (Hyatt et al., 2019). However, the specific mechanisms underlying these processes require further investigation.

Beyond the previously mentioned findings, water TT has been shown to have a beneficial impact on SCI in rats. Specifically, TT can enhance the expression of the BDNF/TrkB-CREB signaling pathway, ameliorate BSCB injury, and facilitate functional recovery. This effect can be inhibited by ANA-12, a specific inhibitor of TrkB, without influencing the normal functionality of blood vessels and BSCB in non-SCI rats, further validating that the neuroprotective mechanism of TT acts through the upregulation of endogenous BDNF expression and activation of downstream signaling pathways (Li et al., 2019, 2020b; Ying et al., 2020). Additionally, the influence of treadmill exercise on neuronal regeneration demonstrates gender-dependent differences. Females respond more prominently to short-term high-speed interval training, whereas males exhibit better responses to continuous low-speed training. This gender disparity may be attributed to the pivotal role of estrogen signal transduction, which modulates the enhancing effect of treadmill exercise on motoneuron regeneration by interacting with BDNF and androgen (Acosta et al., 2017; Otzel et al., 2018). Exercise training may also modulate estrogen signaling and influence muscle metabolism and growth through mechanisms such as increased estrogen secretion or enhanced receptor sensitivity. Moreover, exercise promotes bone formation by regulating the Wnt signaling pathway, while estrogen contributes to bone metabolism by modulating the receptor activator of nuclear factor κB ligand/osteoprotegerin system, thereby inhibiting bone resorption and facilitating bone formation. Both exercise training and estrogen have been shown to increase the population of motor neurons involved in axonal regeneration, with estrogen signaling being a prerequisite for exercise-induced axonal regeneration (Otzel et al., 2018; Shams et al., 2021). Lastly, analyses of the functional electrical stimulation of lower limb circulation or arm training in men with chronic complete SCI revealed that both methods significantly affected the protein expression levels of glucose transporter 4 (GLUT-4), AMPK and PGC-1α in right vastus lateralis and triceps muscles, further emphasizing the importance of exercise in the rehabilitation of spinal cord injury (Gorgey et al., 2017).

#### Effects of exercise training on protein signal transduction

The role of exercise training in the restoration of neurological function in SCI rat models and its association with protein signal transduction is a focal area of current research. Through exploratory studies, Liu et al. (2012, 2023) investigated the effects of exercise on the enhancement of motor function and the optimization of spinal cord morphology in SCI rats, revealing the intricate relationship between exercise and protein signal transduction. The experimental results demonstrated that, following 4 weeks of body-weight-supported treadmill training, rats in the training group not only exhibited significant improvements in spasticity but also remarkable enhancements in motor function, with an optimization of morphology in the spinal cord and gastrocnemius muscle (Li et al., 2022a). Neural mechanistic work indicated that exercise training reduced the expression of transforming growth factor beta 1 (TGF-β1) and hypoxia inducible factor 1 alpha (HIF-1α) in the spinal cord of SCI rats while increasing the levels of the neuron-specific marker MAP1B, neuron-specific enolase, and vimentin (Li et al., 2022a). More importantly, exercise training can inhibit the expression of Nogo-NgR signaling pathway proteins, including Nogo-A, NgR, and leucine-rich repeat and immunoglobulin-like domain containing Nogo receptor interacting protein 1. This inhibition reduces axon growth inhibition and promotes neuronal axon repair and neurite growth (Li et al., 2022a; Ji et al., 2023). This suggests that exercise training may provide an effective means for the recovery of neurological function after SCI by inhibiting the Nogo-NgR signaling pathway.

Furthermore, a study has shown that exercise training-induced BDNF-TrkB signaling can affect the expression of glutamic acid decarboxylase 65/67 (GAD-65/67) and K^+^-Cl^–^ cotransporter 2 by regulating downstream signaling molecules such as Ras/ERK and PLC-1. GAD-65/67 catalyzes the conversion of glutamate to γ-aminobutyric acid (GABA), while KCC2 maintains the inhibitory postsynaptic potential of GABAergic neurons (Liet al., 2020c). Experiments have demonstrated that exercise training can upregulate the expression of GAD-65/67 and KCC2 in the spinal cord after SCI, thereby optimizing the function of GABAergic inhibitory neural circuits and reducing the excessive excitability and spasticity of neurons (Beverungen et al., 2020; Bilchak et al., 2021; Liet al., 2022a). In addition to BDNF-TrkB signal transduction, exercise training can also increase the expression of neurotrophin-3 (NT-3) and NT-4 in the spinal cord of SCI rats, and upregulate the expression of glial cell-line-derived neurotrophic factor (GDNF) and vascular endothelial growth factor (VEGF). These factors have similar biological effects as BDNF and jointly promote neuronal survival and axon regeneration (Huangand Reichardt, 2001; Gómez-Pinillaet al., 2007; Keefeet al., 2017).

The benefits of exercise training on nerve recovery after SCI are not limited to the regulation of protein signal transduction. Experiments have also found that exercise training can promote the remodeling of the central pattern generator (CPG) and local neural circuits after SCI (Barrière et al., 2008; Molinari, 2009; van Hedel and Dietz, 2010). At the same time, physical activity dynamically regulates adult neurogenesis through acetylcholine and GABA neurotransmitters. CPG V2a interneurons, as key mediators of exercise stimuli, promote the proliferation and neurogenesis of neural stem/progenitor cells, closely linking motor function with neurogenesis. Cholinergic V2a spinal interneurons can interact with neural stem/progenitor cells through direct and indirect connections to induce their proliferation (Chang et al., 2021). This process not only links motor network activity to spinal neurogenesis but also provides proof for the non-motor/non-neuronal function of the CPG, providing a new perspective on the internal adaptation mechanisms of the spinal cord after training.

#### Regulation of epigenetics by exercise training

Exercise training affects gene expression by regulating DNA methylation and histone modifications, which play vital roles in the process of nerve regeneration. Specifically, regular exercise training can reduce the methylation level of specific genes, thereby upregulating the expression of the neurotrophic factor BDNF. This process is closely related to the demethylation of the BDNF promoter region, which significantly enhances the transcriptional activity of BDNF. In addition, treadmill training can not only promote the formation of myelin sheaths but also enhance the activity of the Na^+^/K^+^ pump by reducing the expression of chondroitin sulfate proteoglycans (CSPGs), thereby optimizing axon structure and providing the necessary energy and structural support for the safe growth of nerves (Ilha et al., 2011). These changes provide favorable conditions for neuronal plasticity in the corticospinal tract. It is worth noting that genes associated with DNA methylation and hydroxymethylation are activated after treadmill exercise, further supporting the positive impact of exercise on nerve regeneration.

The development and function of mammalian brains are finely regulated by DNA methylation-dependent epigenetic mechanisms (Li et al., 2011). Moreover, specific histone modifications, such as H3K27me3 and H3K9ac, play crucial roles in nerve repair processes after SCI (Xu et al., 2023a). Exercise training has been found to alter chromatin structure and promote nerve regeneration by increasing histone acetylation levels and reducing their methylation status (Murach et al., 2021; Ghosh et al., 2022). Non-coding RNAs, particularly microRNAs, also play an important role in epigenetic regulation. Researchers demonstrated that exercise training can regulate the expression of specific microRNAs, such as miR-29a and miR-21, which are involved in nerve regeneration and repair through apoptosis and proliferation signaling pathways (Li et al., 2020a; Wan et al., 2023). Notably, exercise has been shown to upregulate the concentration of miR-21 circulating in the blood after SCI, exerting a protective effect on neuronal damage. Further research has revealed that exercise-induced miR-21 upregulation protects neurons by inhibiting the expression of the pro-apoptotic gene programmed cell death 4 (PDCD4), which aligns with a previous study that reported the negative regulation of PDCD4 by miR-21 (Li et al., 2020a). Therefore, exercise provides a novel epigenetic regulatory mechanism for SCI treatment by modulating the expression of miR-21 and its target PDCD4.

It should be noted that the state of the nervous system can, in turn, modulate the effect of exercise training. The genetic basis and epigenetic background of an individual may affect neural recovery ability after exercise training. Thus, understanding the relationship between exercise training and epigenetic regulation is of great significance for the development of personalized rehabilitation strategies after SCI.

### Effects of exercise training on the microenvironment of spinal cord injury neurons

For greater clarity, the effects of exercise training on the microenvironment of neuron growth are shown in **Additional Table 2**.

**Additional Table 2:** Effects of exercise training on the microenvironment of neuron growth

Topic	Species	Form of motion	Regulatory mechanism	Result	Reference
Effects of exercise training on inflammation	Rat	MIEtraining (5 m/min 2 times/d, 30 min/time)	Inhibits the NF-κB pathway; reduces HMGB1, TLR4, IL-1β, IL-6, TNF-α, and NF-κB levels	Improves functional recovery; weakens the inflammatory response	Sun et al., 2018
	Man	Acute submaximal aerobic exercise (60 % PPO for 30 min of arm cycle exercise)	Regulates the distribution of white blood cell subsets (Upregulates CD3^+^, CD4^+^, CD3^-^/CD56^+^, and downregulates CD16^+^/ CD14^dim^ monocytes)	Reduces inflammatory response; enhances immune function.	Jackson et al., 2022
	Rat	HIE training	Increases Treg cells and IL-10 production; modulates immune response	Changes in the immune response contribute to recovery	Steensberg et al., 2003; Handzlik et al., 2013
	Rat	Sports training	Increases the level of IL-10; raises the pain threshold; changes the inflammatory response from M1 type to M2 type	Enhances immune regulation; increases pain thresholds	Vidal et al., 2012
	Rat	Submaximal aerobic exercise	Acutely affects cytokines, neuropathic pain, and emotional states	Improves emotional state; reduces neuropathic pain.	Neefkes-Zonneveld et al., 2015; Todd et al., 2021
Effects of exercise training on oxidative stress	Rat	Aerobic exercise	Moderates exercise stabilizes or reduces MDA levels, and trained individuals show lower oxidative stress	Balances oxidative stress; improves recovery	Knez et al., 2007; Teixeira et al., 2009; Lozinski et al., 2021; Weng et al., 2024
	Rat	Endurance exercise	Increases SOD and GPx activity; balances oxidative and antioxidant activity	Recovers motor function; dynamically regulates neurogenesis	Powers and Jackson, 2008; Radak et al., 2008; Armada da-Silva et al., 2013
	Man	Different types of exercise training (aerobic, anaerobic, combined training)	Long-term aerobic training reduces the oxidation of low-density lipoprotein, while a single exercise to exhaustion causes an increase in oxidative stress markers	Protocol-dependent effects on oxidative stress levels	van Duijnhoven et al., 2010; Goldhardt et al., 2019
	Man	FES and aerobic Training	Strength may not be valid	Increasing antioxidant capacity may not be effective	Maugeri et al., 2023
	Man	Upper limb basketball	Exercise affects blood flow and NO production, and mitochondrial activity produces oxidative stress, which is evaluated by TBARS	The TBARS values of athletes and nonexercise patients are significantly lower than those of the control group, and the plasma NO levels are not significantly different between the groups	Ordonez et al., 2013; Mitsui et al., 2017; Maugeri et al., 2023

ACE: Arm cycling ergometry; BDNF: brain-derived neurotrophic factor; BSCB: blood-spinal cord barrier; CFA: complete Freund's adjuvant; FES: functional electrical stimulation; GLUT-4: glucose transporter type 4; HIE: high-intensity exercise; HIF-1α: hypoxia-inducible factor-1 alpha; IL-1β: interleukin-1 beta; IL-10: interleukin-10; IL-6: interleukin-6; MAP1B: microtubule-associated protein 1B; MBP: myelin basic protein; MIE: moderate-intensity exercise; MMP-2/9: matrix metalloproteinase-2/9; NSE: neuron-specific enolase; NO: nitric oxide; PGC1α: peroxisome proliferator-activated receptor gamma coactivator 1-alpha; PPO: peak power output; ROS: reactive oxygen species; TBARS: thiobarbituric acid reactive substances; TGF-β1: transforming growth factor-β1; TJ/AJ: tight junction/adhesion junction; VIM: vimentin.

#### Effects of exercise training on inflammation

Exercise training has a central role in modulating the inflammatory response after SCI (Walsh et al., 2023). A study on preclinical SCI models showed that exercise can effectively inhibit the expression of pro-inflammatory cytokines. Sun et al. revealed that moderate-intensity exercise significantly reduced IL-1β levels, and this was accompanied by a significant improvement in functional recovery and attenuation of the NF-κB pathway signaling (Sun et al., 2018). Tang et al. (2022) also ascertained that TT reduced NF-κB signal transduction and IL-1β expression in a rat contusion model. In SCI patients, exercise training also shows a positive effect on inflammation. Specifically, one study observed that 30 minutes of acute submaximal aerobic exercise effectively regulated the distribution of white blood cell subsets in SCI patients, increased the number of CD3^+^, CD4^+^, and CD3^–^/CD56^+^ cells with effector function, and reduced the number of pro-inflammatory CD16^+^/CD14^dim^ monocytes during recovery (Jackson et al., 2022). This suggests that exercise may reduce the potency of the inflammatory response after SCI by regulating white blood cell subsets.

Although the IL-6 released during high-intensity exercise is often regarded as a pro-inflammatory cytokine, the authors of Steensberg et al. (2003) showed that it also has anti-inflammatory effects. IL-6 is released from contractile skeletal muscle and triggers an increase in the circulating levels of the anti-inflammatory cytokine IL-10 and IL-1 receptor antagonist. Similar increases in IL-6 levels are observed during exercise training in human patients with SCI and are not accompanied by an increase in the levels of the pro-inflammatory cytokine TNF-α. This suggests that exercise training does not induce an acute inflammatory response after SCI but may promote recovery by regulating the expression of cytokines such as IL-6 (Walsh et al., 2023).

The effect of exercise on inflammation after SCI is complex and changeable and is regulated by many factors, including exercise type, duration, and intensity. A study has shown that submaximal aerobic exercise has an acute effect on the level of inflammatory cytokines, neuropathic pain, the emotional state, and awakened state of SCI individuals (Todd et al., 2021). Under different conditions of long-term physical activity and short-term acute exercise, the trend in IL-6 concentration changes in patients with quadriplegia and paraplegia after exercise showed significant differences (Neefkes-Zonneveld et al., 2015). This further confirmed the significant effect of exercise intensity on inflammatory cytokine changes (especially IL-6). In addition, exercise training has been shown to have the ability to increase the production of the anti-inflammatory cytokine IL-10 and thus contribute to recovery after SCI. Handzlik et al. (2013) pointed out that high-intensity exercise training can increase the number of Treg cells in healthy mice, thereby promoting the production of IL-10 and triggering changes in immune response. In an SCI model, exercise training not only increased the level of IL-10 but was also accompanied by an increase in pain threshold (Vidal et al., 2012). This further supports the positive role of exercise in the regulation of inflammation after SCI.

In summary, future research should explore the effects of specific exercise intensities, types, and times on inflammatory cascades, neuropathic pain, and emotional responses in SCI patients. Additionally, the potential benefits of using inflammatory cytokines as biomarkers for predicting exercise responsiveness should be verified. This will provide a more accurate and effective exercise intervention strategy for the rehabilitation of SCI patients and promote the recovery of overall patient function.

#### Effects of exercise training on oxidative stress

There is a complex and crucial interaction network connecting exercise training and oxidative stress, especially in the exercise training of SCI patients (Rodrigues et al, 2023; Wouda et al., 2023). Exercise training, especially strenuous physical activity, may cause oxidative stress and related inflammation, which is a key factor that must be considered when determining the optimal intensity of SCI exercise training. Aerobic exercise has been shown to increase free-radical concentrations in rodents and humans (Lozinski et al., 2021). Malondialdehyde (MDA), a highly reactive product of lipid peroxidation, is often used as a biomarker for assessing oxidative stress (Weng et al., 2024). A number of works, such as those by Knez et al. (2007) and Teixeira et al. (2009), have shown that the level of MDA increases significantly after exercise, but the levels of MDA in rats show a decreasing trend or no significant change in response to moderate exercise. In addition, the increase in plasma MDA levels after exercise in well-trained individuals is usually less dramatic than that in untrained individuals, and a lower concentration of oxidative stress markers and higher antioxidant enzyme activity at rest are seen in well-trained individuals (Knez et al., 2007; Teixeira et al., 2009).

The effect of exercise training on SCI patients is also complex. On one hand, exercise can enhance the strength of paralyzed muscles, promote the recovery of motor function, and dynamically regulate neurogenesis in the intact spinal cord (Santos et al., 2022; Shackleton et al., 2023). For instance, in a rodent model of SCI, endurance exercise promoted axonal regeneration, myelin formation, and nerve fiber recovery through multiple mechanisms (Armada-da-Silva et al., 2013). On the other hand, exercise may also lead to an increase in oxidative stress levels in SCI patients, but this increase is usually balanced by a gradual activation of antioxidant activity. For example, the increased production of antioxidant enzymes, such as superoxide dismutase and glutathione peroxidase, helps to train the body’s ability to rebalance the ratio of antioxidant activity to oxidative activity (Powers and Jackson, 2008; Radak et al., 2008).

However, the effects exercise on oxidative stress in SCI patients differ with different types of training regimens. For example, aerobic, anaerobic, and aerobic-anaerobic combined training types produce different metabolic responses. Long-term aerobic training can reduce the oxidation of low-density lipoprotein, while a single exercise to exhaustion may lead to an increase in oxidative stress markers (Goldhardt et al., 2019). The duration and intensity of exercise training is also a key factor affecting the level of oxidative stress. Moderate exercise may help reduce oxidative stress levels, while strenuous and prolonged exercise may exacerbate oxidative stress (van Duijnhoven et al., 2010). In SCI patients, the regulatory effect of exercise training on oxidative stress is affected by the daily physical activity and initial health levels of the individual. The concentration of antioxidant markers in active subjects increases after exercise, indicating that the effect of exercise on oxidative stress is protocol-dependent. By contrast, sedentary SCI subjects appear to have higher levels of lipid peroxides at rest and in response to a single exercise test. In addition, the combined application of swimming training and microbiota transplantation led to a higher intervention efficiency after SCI, and more effectively enhanced physical mobility and improved neurological function compared to a single intervention (Li et al., 2024c). *Grammostola spatulata* mechanotoxin 4 (GsMTx4) is a mechanosensitive ion channel inhibitor extracted from spider venom. In the rat T10 SCI model, combined treatment with GsMTx4 and body-weight-supported treadmill training was more effective than either treatment alone. This combination reduced calcium ion concentrations, improved lysosomal function, enhanced autophagy, decreased apoptosis and demyelination, and significantly improved motor function in the rats (Li et al., 2024c).

Despite these complexities, exercise training can affect oxidative stress management in SCI patients in a variety of ways. For example, the chronic effects of functional electrical stimulation combined with aerobic training programs have been studied. However, the results showed that the intensity of this training method may not be effective in improving the antioxidant capacity of SCI subjects (Maugeri et al., 2023). Moreover, other studies (Ordonez et al., 2013; Mitsui et al., 2017; Maugeri et al., 2023) have found that different types of exercise (e.g., arm crank aerobic exercise, wheelchair basketball, wheelchair half-marathon) may have different effects on oxidative stress levels in SCI patients. These differences may be related to the duration, intensity, and type of exercise training, as well as the initial health and daily physical activity levels of the subjects. Notably, regular physical exercise can regulate cellular redox status by upregulating metabolism and/or directly activating the enzymes that produce ROS. Thus, regulation of the enzyme antioxidant system and resistance to oxidative damage are increased (Garbeloti et al., 2016). This adaptive change not only helps to balance the level of oxidative stress but may also have a positive impact on secondary consequences, such as neuroplasticity, motor function recovery, and chronic inflammation, in SCI patients.

## Repair and Regeneration of Glial Cells After Spinal Cord Injury

### Repair and regeneration of astrocytes after spinal cord injury

#### Pathological changes of astrocytes after spinal cord injury

**Figure 4:**
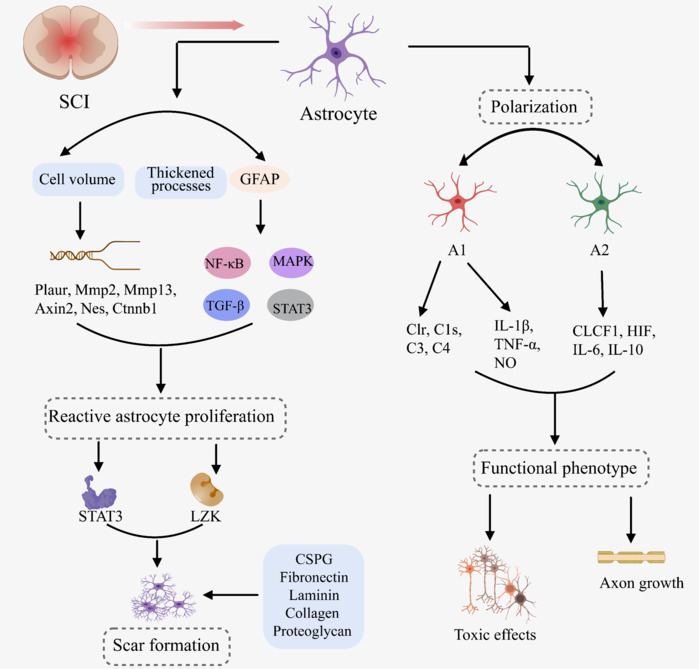
Pathological mechanism of astrocytes after SCI. Created with MedPeer (medpeer.cn). The pathological changes in astrocytes after SCI involve complex and multifaceted processes, including reactive glial cell proliferation, glial scar formation, polarization into different functional phenotypes, and significant changes in their functional roles. CLCF1: Cardiotrophin-like cytokine factor 1; Ctnnb1: catenin beta 1; CSPG: chondroitin sulfate proteoglycans; GFAP: glial fibrillary acidic protein; HIF: hypoxia-inducible factor; IL-1β: interleukin-1 beta; IL-6: interleukin-6; IL-10: interleukin-10; LZK: leucine zipper kinase; MAPK: mitogen-activated protein kinase; Mmp2: matrix metalloproteinase 2; Mmp13: matrix metalloproteinase 13; Nes: nestin; NF-κB: nuclear factor kappa-light-chain-enhancer of activated B cells; NO: nitric oxide; Plaur: plasminogen activator, urokinase receptor; SCI: spinal cord injury; STAT3: signal transducer and activator of transcription 3; TGF-β: transforming growth factor-beta; TNF-α: tumor necrosis factor-alpha.

The pathological changes in astrocytes after SCI involve complex and multi-dimensional processes involving glial scar formation, reactive glial cell proliferation, polarization, and functional phenotype changes (**Figure 4**). These changes not only reveal the central role of astrocytes in the response to injury but also provide important targets and ideas for exploring treatment strategies for SCI. The following is a detailed description of the pathological changes in astrocytes after SCI.

*Reactive glial cell proliferation*

After SCI, astrocytes respond quickly to injury signals and initiate the process of reactive glial cell proliferation (Li et al., 2020b). This process is accompanied by significant morphological changes in astrocytes, including increased cell volume, thickened protrusions, and a significant increase in the expression level of intermediate filament proteins (e.g., GFAP). These changes collectively define the characteristics of reactive astrocytes (Althammer et al., 2020; Yang et al., 2020a). Reactive astrocytes also undergo changes in their gene expression profiles, involving a variety of genes such as *Plaur*, *Mmp2*, *Mmp13*, *Axin2*, *Nes*, and *Ctnnb1*, which further regulate cell proliferation and biochemical characteristics. At the molecular mechanism level, a series of signaling pathways, such as NF-κB, STAT3, TGF-β, and MAPK pathways, are rapidly activated in the acute phase after injury and continue to regulate the reactive proliferation of astrocytes. The activation of these pathways is a response to mechanical damage signals and is also regulated by paracrines from other cell types such as microglia and immune cells (Huang et al., 2024; Reyes and Mokalled, 2024). Notably, reactive astrocyte proliferation is a continuous process that is stimulated by a variety of internal and external factors. These factors lead to a series of changes in the transcriptional profile of astrocytes, which in turn enable the cells to acquire new biochemical, morphological, metabolic, and physiological states (Lu et al., 2024; O’Shea et al., 2024).

*Scar formation*

With the progression of reactive glial cell proliferation, astrocytes accumulate and proliferate around the injury site, gradually forming a dense physical barrier, namely a glial scar. This process may be mediated by STAT3 signal transduction and leucine zipper kinase expression, ensuring the stability and persistence of newly formed astrocyte structures (Wanner et al., 2013). Moreover, astrocytes and fibroblasts co-secrete a variety of extracellular matrix molecules, such as CSPG, fibronectin, laminin, collagen, and proteoglycan, molecules that provide the glial scar with shape, stability, and mechanical strength (Ou et al., 2023; Tamaru et al., 2023). The formation of a glial scar plays a dual role in the pathological process of SCI. On the one hand, it limits the expansion of the inflammatory core and the further expansion of the fibrotic core, thereby protecting undamaged nerve tissue from secondary damage. On the other hand, the dense structure of the scar and the enrichment of inhibitory molecules (e.g., CSPG) constitute physical and chemical barriers to axonal regeneration (Jeong et al., 2021; Gong et al., 2023; Bagheri et al., 2024).

*Polarization*

After SCI, astrocytes also show different polarization states that are closely related to their functional diversity in injury repair. It has been reported that astrocytes can polarize into A1 (pro-inflammatory) and A2 (anti-inflammatory/neuroprotective) phenotypes. A1 phenotype astrocytes express high levels of complement system cascade genes (e.g., *C1r*, *C1s*, *C3*, and *C4*) and pro-inflammatory factors (e.g., IL-1β, TNF-α, and NO) (Pang et al., 2022). These factors have toxic effects on neurons and oligodendrocytes, exacerbating nerve tissue damage. In contrast, A2 phenotype astrocytes secrete cardiotrophin-like cytokine factor 1, HIF, IL-6, IL-10, and thrombospondin, which can promote neuronal survival, axon growth, and nerve repair (Wang et al., 2023a; Li et al., 2024a). The polarization state of astrocytes is directly regulated by damage signals, but it is also affected by other cell types such as microglia and immune cells. These complex intercellular interactions further increase the complexity of the pathological process of SCI and therapeutic challenges.

*Functional phenotypes*

After SCI, significant changes in the functional phenotypes of astrocytes occur. They change from supporting cells that maintain the normal homeostasis of the CNS to dynamic cells that actively participate in the injury response and repair process. Astrocytes release a variety of cytokines, chemokines, growth factors, toxic amino acids, extracellular matrix molecules, and proteoglycans (Akram et al., 2022). These form a complex microenvironment at the site of injury that has a profound impact on various cell types, including neurons, oligodendrocyte precursor cells, and microglia. For example, growth factors, such as BDNF and GDNF, released by astrocytes help promote neuronal survival and axonal growth (Li et al., 2022b). Moreover, the release of toxic amino acids, such as GABA and glutamic acid, may have toxic effects on neurons and aggravate the death and dysfunction of neurons. Astrocytes are also actively involved in the regulation of neuroinflammation. They affect the processes and outcomes of the inflammatory response by secreting inflammatory mediators such as TNF-α, IL-6, and IL-1β (Yang et al., 2024; Zhang et al., 2024b).

#### Effects of exercise training on the repair and regeneration of astrocytes after spinal cord injury

For greater clarity, we have added a table to explain how exercise training mediates the repair effect of astrocytes on SCI (**Additional Table 3**).

**Additional Table 3:** Exercise training mediates the repair effect of astrocytes on SCI

Species	Form of motion	Regulatory mechanism	Result	Reference
Rat	Swimming training	Downregulates GFAP expression	Improves motor recovery; reduces astrocyte activation	Ouyang et al., 2023
Rat	Running exercise	Upregulates BrdU^+^/GFAP^+^ cells	Enhances astrocyte density and neurogenesis	Li et al., 2023
Rat	Treadmill + Sox2 reprogramming	Converts astrocytes to neurons	Recovers function; reduces glial scar density	Yang et al., 2020
Rat	Treadmill (30 min/d, for 3-6 wk)	Upregulates GFAP and VEGF	Enhances angiogenesis and neurovascular coupling	Li et al., 2005; Latimer et al., 2011
Rat	Water treadmill training (30°C, 10-15 m/min, 5 min/time, a total of 3 times, interval of 5 min, for 7-14 d)	Upregulates VEGF, p120-Catenin, β-Catenin, ZO-1, Occludin and Claudin-5; downregulates MMP-2/9	Reduces BSCB damage; improves tissue and functional recovery; promotes angiogenesis	Ying et al., 2020
Rat	Weight-bearing walking training (8 m/min, 10 min/time, 1 time/d, 6 d/wk)	Downregulates TGF-β1, HIF-1α, Nogo-A, NgR and LINGO-1; upregulates MAP1 B, NSE and VIM	Enhances axonal repair, neurite growth, and motor function	Ji et al., 2023
Rat	The combined G-CSF and treadmill exercise (30 min/d, 5 d/wk, for 4 wk)	Upregulates VEGF and BDNF; downregulates GFAP	Reduces syringomyelia; inhibits astrocyte activation; reduces glial scar formation; promotes nerve repair	Steensberg et al., 2003; Handzlik et al., 2013; Park et al., 2020
Rat	Treadmill training (20-30 m/min, 15-60 min/time, exercise intensity gradually increased, 5 d/wk, for 3 wk)	Upregulates HSP72	Reduces astrocyte apoptosis and gray matter contusion	Chang et al., 2021
Rat	Treadmill training (6.3 m/min-8.6 m/min, 5 d/wk, for 4 wk)	Activates CPG; regulates local microenvironment	Reduces the syringomyelia; improves motor function; promotes axon regeneration	Griffin et al., 2020
Rodent	Plantar weight-bearing training	Degrades CSPG synthesis; remodeling ECM	Reduces cavitation; promotes axon regeneration	Hayashibe et al., 2016

ACh: Acetylcholine; BDNF: brain-derived neurotrophic factor; BrdU: bromodeoxyuridine; BSCB: blood-spinal cord barrier; CPG: central pattern generator; CSPG: chondroitin sulfate proteoglycan; EAAT2: excitatory amino acid transporter 2; FMT: fecal microbiota transplantation; GABA: gamma-aminobutyric acid; G-CSF: granulocyte colony-stimulating factor; GFAP: glial fibrillary acidic protein; GsMTx4: grammostolaspatulatamechanotoxin 4; HIF-1α: hypoxia inducible factor-1 alpha; HSP 72: heat shock protein 72; MBP: myelin basic protein; MMP-2/9: matrix metalloproteinase-2/9; NgR: Nogo-66 receptor; NSE: neuron-specific enolase; NSPC: neural stem/progenitor cell; OL: oligodendrocyte; OPC: oligodendrocyte precursor cell; Sox2: SRY-box transcription factor 2; TGF-β1: transforming growth factor-31; TT: treadmill training; VEGF: vascular endothelial growth factor; VIM: vimentin; ZO-1: zonula occludens-1.

*Exercise training regulates the activation and morphological changes of astrocytes*

After SCI, the effect of exercise training on astrocyte activation results in multiple effects. A recent study showed that swimming training can significantly reduce the expression of GFAP, a marker protein of astrocytes after SCI, and has a positive effect on the recovery of motor function (Ouyang et al., 2023). However, in neurodegenerative diseases, running exercise has been shown to enhance the density of astrocytes and promote the formation of new astrocytes, as evidenced by a significant increase in the number of bromodeoxyuridine (BrdU)^+^/GFAP^+^ cells (Li et al., 2023). These changes appear to strengthen the functions of astrocytes that support and protect neurons. Notably, this is in sharp contrast to the mechanism by which the above-mentioned exercise training inhibits the activation of astrocytes after SCI. The difference may be due to the type of disease, the exercise regimen, and the time of intervention. The literature suggests that we need to fully consider the complexity of these factors when studying the effects of exercise training on astrocyte activation.

With the help of an adeno-associated virus vector, Tai et al. (2021) used the single transcription factor Sox2 to reprogram some astrocytes into neurons after SCI in adult mice. Yang et al. (2020) found that this astrocyte reprogramming can not only replenish lost neurons but also moderately reduces the density of glial scars without destroying their integrity. Combined with treadmill training, Sox2-induced astrocyte reprogramming significantly improved functional recovery. This suggests that exercise training may promote neural repair and functional recovery by affecting the activity and function of astrocytes. However, a study by Khristy et al. (2009) also showed that the effect of exercise training on astrocytes may be relatively small, or needs to be combined with other factors to achieve a significant effect. This specific effect may be closely related to the type and function of neurons and the degree of participation in specific motor tasks. For instance, during gait training, the tibialis anterior muscle serves as the primary ankle flexor, with its motor neurons being frequently activated. This activity promotes the recovery of γ2 subunit expression, which may indirectly influence the activation state of astrocytes.

The effect of exercise training on the morphological characteristics of astrocytes is also remarkable (Loprinzi, 2019; Lundquist et al., 2019). Moderate exercise training helps to maintain the normal morphological structure of astrocytes and effectively prevent excessive morphological changes due to injury stimulation (Zheng et al., 2024). Scholars (Saur et al., 2014; Yamaguchi et al., 2023; Li et al., 2024d) have shown that moderate exercise training can increase the degree of astrocyte bifurcation, induce more star-shaped cells, and increase the length of protrusions. These effects contribute to more wide-scale contact and interactions between astrocytes and neurons. However, it is worth noting that these morphological changes have regional specificity, and their specific mechanisms remain to be further studied.

*Exercise training affects astrocyte polarization and inflammatory mediator release*

After SCI, astrocytes respond quickly by releasing a large number of inflammatory mediators, triggering a series of complex reactions (Zhang et al., 2023; Wang et al., 2024). Wheel running exercise was reported to reduce the expression of IL-17 and increase the expression of IL-10 and Nrf-2 in animal models. Moreover, the activation of Nrf-2 can inhibit the expression of NF-κB, IL-1β, IL-6, and other inflammatory mediators, thereby reducing the strength of the inflammatory response (Saffar et al., 2020; Zheng et al., 2024). However, differences in experimental models, exercise intensity, and methods may lead to differences in the polarization of astrocytes and the release of inflammatory mediators (Sun et al., 2018; Nakanishi et al., 2021). In a study of related neurological disorders (Nakanishi et al., 2021), regular low-intensity running at a constant speed of 2.4 m/min was shown to significantly inhibit the protein levels of neurotoxic A1 phenotype astrocytes in mice. This activity also reduced the release of inflammatory mediators, such as C3, thereby decreasing neuroinflammation in the hippocampus. Furthermore, moderate exercise can cause astrocytes to convert from an A1 phenotype to an A2 phenotype; reduce the levels of inflammatory mediators such as IL-1α, TNF, and C1q; and promote the release of TGFβ (Jiang et al., 2021). These phenomena further emphasize the key roles of exercise intensity and mode in regulating the direction of astrocyte polarization.

In contrast, after 2 hours of high-intensity acute swimming training, astrocytes in the spinal dorsal horn of mice were activated, and the release of inflammatory mediators such as TNF-α and IL-1β increased. After intervention with astrocyte inhibitors, the expression of exercise-induced inflammatory mediators was effectively reduced. This suggests that high-intensity exercise may lead to the activation of astrocytes and an enhancement of the inflammatory response, which is in stark contrast to the regulatory effect of exercise on astrocytes observed in the previous study. However, in an experimental autoimmune encephalomyelitis (EAE) model, high-intensity swimming training reduced the levels of IFN-γ and IL-17 and increased the levels of serum TGF-β and IL-10, thereby alleviating clinical symptoms. Whereas moderate intensity swimming training had no significant effect on inflammation or pathological symptoms (Xie et al., 2019).

Notably, the choice of exercise intensity is crucial for SCI patients. High-intensity exercise may help regulate the secretion of glucocorticoids, catecholamines, and other substances via changes to sympathetic nervous system and hypothalamic-pituitary-adrenal axis activity. In turn, it affects the balance of proinflammatory and anti-inflammatory cytokines, which may indirectly affect the polarization of astrocytes (Saffar et al., 2020). There is limited literature specifically addressing how exercise intensity influences astrocyte polarization and the release of inflammatory mediators in SCI models. However, research has once again confirmed that exercise training exerts a common regulatory effect on astrocytes across different disease models. This effect manifests through alterations in the polarization state of astrocytes and the release patterns of inflammatory mediators, which in turn help improve disease-related symptoms. These findings provide a valuable reference for future research on SCI.

*Exercise training improves the metabolic function of astrocytes*

Astrocytes maintain normal physiological activities and actively participate in injury repair, making them an integral player in the support mechanisms of good metabolic function. Exercise training has a significant effect on optimizing the metabolic state of astrocytes after SCI. Studies have shown that exercise can effectively regulate the activity of energy metabolism-related transporters and enzymes (e.g., EAAT2 and GS) in astrocytes, promote the efficient synthesis and rational utilization of intracellular energy substances, and further improve neural plasticity (Xue et al., 2022; Sleijser-Koehorst et al., 2023). Specifically, exercise can significantly increase the activity of ATP synthase in the mitochondria of astrocytes, enhance the aerobic respiration ability of cells, and provide a stable and sufficient energy source for cell repair and regeneration (Matsui et al., 2017). At the molecular level, exercise activates the AMPK signaling pathway, upregulates the expression of key genes such as GLUT-1 and hexokinase, and promotes the glucose uptake and utilization efficiency of astrocytes (Wang et al., 2002; Allen and Messier, 2013; Xue et al., 2024). In addition, exercise also affects the activity of enzymes related to lipid metabolism in astrocytes, effectively regulates the balance of lipid metabolism, and ensures the normal structure and function of cells (Santin et al., 2011). It is worth noting that exercise-induced HSP-72 expression plays an important role in neuroprotection after SCI. HSP-72 may directly protect astrocytes from injury stress and maintain the stable structure and function of intracellular proteins (Santin et al., 2011). It may also be involved in regulating the metabolic process of astrocytes and enhancing their energy production and substance synthesis to support their increased activity during SCI repair. Moreover, the interactions of HSP-72 with other intracellular signaling molecules (e.g., Akt and other protein kinases) may activate the intracellular anti-apoptotic signaling pathway (Fauconneau et al., 2002), reduce the apoptosis of astrocytes, and further promote their survival and repair functions.

*Exercise training promotes angiogenesis after spinal cord injury*

Angiogenesis plays a crucial role in the repair process after SCI, and astrocytes are significant in mediating the mechanism of exercise-induced angiogenesis (Ying et al., 2020). In a study of adult male Sprague–Dawley (SD) rats that performed 30 minutes of exercise daily on a treadmill, the number of astrocytes and the expression of GFAP in the spinal cord tissue increased significantly after 3 or 6 weeks. This finding preliminarily revealed the potential association between exercise-induced astrocyte proliferation and angiogenesis. Moreover, it provided a preliminary clue for understanding the interaction between exercise, astrocytes, and angiogenesis (Li et al., 2005). Further research was carried out in middle-aged female C57 BL/6 mice. Specifically, researchers found that aerobic exercise reduced the hypertrophy of astrocytes in the hippocampus—a sign of aging—accompanied by an increase in VEGF levels. This change makes the surface of the blood vessel smoother and the area of the endothelial cell nucleus larger (Latimer et al., 2011). Notably, this positive effect may be due to the fact that exercise reduces the burden of astrocytes and promotes normal neurovascular coupling.

In their study using a rat SCI model, scholars found that water TT enhanced the expression levels of tight junction and adhesion junction proteins and helped to maintain the integrity of the BSCB by promoting BDNF/TrkB-CREB signaling pathway activity (Ying et al., 2021). In view of the fact that astrocytes are an important part of the BSCB, their function may be regulated under the action of TT, thus affecting blood vessels. In addition, TT can also significantly improve the expression of VEGF, promote angiogenesis, and maintain the stability of the BSCB (Ying et al., 2020). Other studies have also demonstrated that exercise training improves motor function in rats with SCI and regulates the expression of TGF-β1 and HIF-1α in spinal cord tissue (Ji et al., 2023; Park et al., 2020). Although these studies did not directly address the role of astrocytes, in view of the core position of astrocytes in SCI repair, we can speculate that exercise training may indirectly enhance vascular repair and regeneration by modulating astrocyte activity, improving the local microenvironment, and reducing nerve damage.

Not only can exercise training activate astrocytes and regulate a variety of factors that are essential for neurovascular integrity (e.g., endothelin-1, VEGF, insulin-like growth factor 1), it also regulates other angiogenesis-related signaling pathways (e.g., Akt and ERK) (Zhang et al., 2022). The activation of the Akt signaling pathway can inhibit the activity of pro-apoptotic protein Bcl-2-associated death promoter and promote the survival and proliferation of endothelial cells. Moreover, the activation of the ERK pathway contributes to endothelial cell migration and tube formation (Kojda and Hambrecht, 2005). These findings together reveal the complex mechanism by which exercise acts in promoting angiogenesis and repair after SCI and provide new perspectives and ideas for future research.

*Exercise training reduces glial scar formation and promotes axonal regeneration*

Astrocytes play crucial roles in tissue repair and regeneration after SCI. After SCI, astrocytes respond quickly, accumulate in the injured area, and form glial scars rich in inhibitory molecules such as CSPG. Notably, this pathological process significantly hinders the regeneration of axons (Curcio and Bradke, 2018). However, as an effective intervention, exercise training effectively regulates this pathological process through a variety of mechanisms.

First, exercise may mitigate the inhibitory effects of the glial scar on axon regeneration by promoting the degradation of CSPG by astrocytes or reducing its synthesis level. Additionally, exercise may regulate the expression of extracellular matrix components and intercellular adhesion molecules (Wang et al., 2014), which in turn influence the migration trajectory and aggregation behavior of astrocytes. This regulation encourages astrocytes to form a relatively loose structure, rather than a dense one at the injury site, thereby creating more favorable spatial conditions for axonal regeneration (García-Alías and Fawcett, 2012). It is worth noting that exercise training can also promote interactions between microglia and astrocytes and jointly regulate the formation of glial scars. These interactions may involve the secretion of factors, such as insulin-like growth factor 1, that promote astrocyte repair and inhibit scar formation. As a result, a synergistic regulatory mechanism may be established between cells to collectively enhance the repair process after SCI (Pereira et al., 2015; Bernardes and de Oliveira, 2018; Ageeva et al., 2024).

In a study of tissue recovery after SCI, plantar weight-bearing forced training conferred a significant effect of reducing cavity formation and enhancing axon regeneration. This finding may be closely related to the fact that exercise activates astrocytes to better support and guide axonal regeneration (Hayashibe et al., 2016). Recently, a study has shown that a combination of exercise training and adeno-associated virus vector-mediated ADAMTS4 gene therapy can further enhance functional improvement after SCI (Griffin et al., 2020). Exercise may enhance the response of astrocytes to CSPG degradation by modulating their activity and optimizing the distribution and transduction efficiency of gene therapy vectors in the spinal cord. This leads to more effective CSPG degradation, a reduction in glial scar inhibition, and the promotion of the repair process after SCI. In a rat SCI model experiment, a combination of treadmill training and granulocyte colony-stimulating factor was found to have a significant effect on the recovery of motor function and reduction of spinal cord cavity size (Park et al., 2020). The combined treatment inhibited the ability of astrocytes to form glial scars and increased the expression of BDNF and VEGF. The above research not only revealed the potential of using a combination of exercise training and other methods for synergistically regulating astrocytes but also the exercises’ ability to reduce the obstruction of glial scars on angiogenesis and promote angiogenesis.

*Exercise training regulates the expression and secretion of nerve growth factor*

As a key target and effector cell, astrocytes play a central role in the motor regulation of nerve growth factor expression. Specifically, exercise training can effectively promote the expression of key nerve growth factors, such as BDNF, GDNF, and fibroblast growth factor (Almeida et al., 2015; Sleijser-Koehorst et al., 2023). At the cellular level, exercise may achieve this regulation by activating specific signaling pathways, e.g., the BDNF, PI3K-Akt, and ERK pathways, in astrocytes. After activation of the PI3K-Akt pathway, Akt is phosphorylated and enters the nucleus, where it binds to a specific sequence in the BDNF gene promoter region and promotes its transcription (Li et al., 2024c; Zhang et al., 2024a, c). Moreover, the activation of the ERK pathway enhances the transcription efficiency of BDNF by phosphorylating the downstream transcription factor CREB (Ying et al., 2021). Exercise may also activate calmodulin-dependent protein kinase and promote the secretion of BDNF by regulating the concentration of calcium ions in astrocytes (Gomez-Pinillaet al., 2012; Joseph et al., 2012; Liu et al., 2023a).

The growth factor GDNF enhances neuronal survival and growth, and exercise training may promote its expression by regulating the activity of transcription factors such as STAT3 (Li et al., 2024b). Activation of the JAK-STAT pathway causes STAT3 to be phosphorylated and enter the nucleus, where it binds to the *GDNF* gene promoter region to enhance its transcription. Exercise may further promote the expression and secretion of GDNF by regulating the intracellular redox state, such as by activating the Nrf2 pathway (Saffar et al., 2020; Maugeri et al., 2023). These nerve growth factors play a key role in repair processes after SCI, not only improving the functional status of neurons but also acting on astrocytes through paracrine or autocrine signals to promote the repair and regeneration of astrocytes. This forms a benign repair cycle that continues to promote the recovery of neurological function after SCI.

### Repair and regeneration of microglia after spinal cord injury

#### Pathological changes in microglia after spinal cord injury

**Figure 5:**
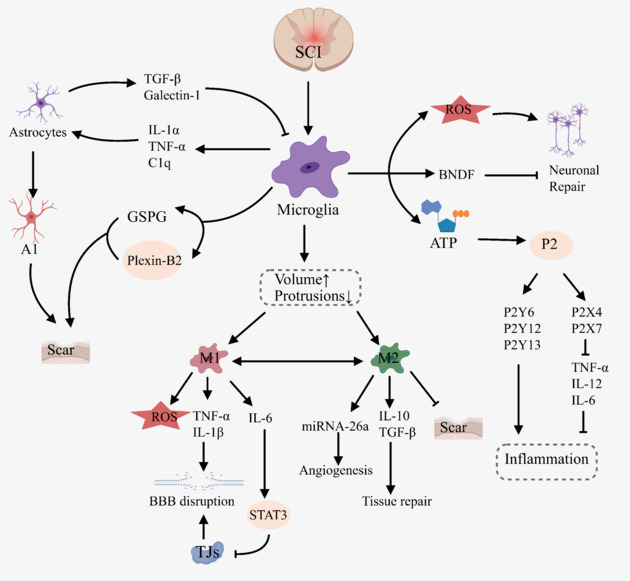
Pathological mechanism of microglia after SCI. Created with MedPeer (medpeer.cn). Microglia undergo dynamic changes in phenotype and function after SCI, playing a central role in the immune response, inflammatory regulation, scar formation, blood‒spinal cord barrier disruption, and complex interactions with neurons, astrocytes, and other cells. These processes collectively drive pathological progression and impact the prospects for repair and recovery. ATP: Adenosine triphosphate; BDNF: brain-derived neurotrophic factor; C1q: complement component 1q; galectin-1: galectin-1; GSPG: glycosaminoglycan sulfate proteoglycans; IL-1α: interleukin-1 alpha; IL-1β: interleukin-1 beta; IL-6: interleukin-6; IL-10: interleukin-10; ROS: reactive oxygen species; SCI: spinal cord injury; STAT3: signal transducer and activator of transcription 3; TGF-β: transforming growth factor-beta; TJS: tight junctions; TNF-α: tumor necrosis factor-alpha.

Microglia exhibit a steady-state phenotype in uninjured spinal cord tissue and actively participate in the development of SCI. Notably, microglia not only play an important role in regulating vascular development but also settle in developing spinal cord tissue during embryonic development, laying the foundation for the normal development of the nervous system. After entering adulthood, microglia play an important role in immune surveillance. Its main function is to ensure the homeostasis of the SCI microenvironment, maintain normal interactions between neurons and astrocytes, and thus ensure the normal functioning of neural circuits (Hellenbrand et al., 2021; Mehl et al., 2022; Sun and Jiang, 2024). Moreover, we provide a diagram of the pathological mechanism of microglia after SCI in **Figure 5**.

*Immune response and inflammatory regulation of microglia after spinal cord injury*

After SCI, microglia are rapidly activated, and the expression of proinflammatory markers and growth factors is upregulated within approximately 2 hours. Its morphology is characterized by increased volume and shortened protrusions, and its function is enhanced by phagocytosis and cytokine secretion, which are key to the early immune response (Xue et al., 2022; Ma et al., 2023; Schreiner et al., 2024). In an SCI model in C57BL/6J mice, the transformation of disease-related microglia begins at 3 dpi, continues to occur at 90 dpi, and then peaks at 7 dpi, reflecting dynamic changes (Timofeeva et al., 2025). The activation of microglia is related to the precise regulation of their proliferation, degree of damage, local microenvironment (cytokine concentration and metabolite level), and specific activation molecules (damage-related molecular patterns) (Ling and Wong, 1993; Pang et al., 2022). Although the traditional M1/M2 phenotype classification has certain explanatory power, it has been unable to fully describe the complexity of microglia. M1 microglia dominate the inflammatory response in the early stage, secrete proinflammatory factors (e.g., TNF-α, IL-1β, and IL-6), and destroy the BSCB. They can also promote leukocyte infiltration and angiogenesis, aggravate neuronal and oligodendrocyte death, and drive the inflammatory cascade (Akhmetzyanova et al., 2018). In addition, M1 microglia can produce ROS, disrupt redox balance, damage cellular components, and cause tissue edema and peroxidation. In contrast, M2 microglia exert anti-inflammatory and repair functions, secrete anti-inflammatory and neurotrophic factors (e.g., IL-10 and TGF-β), inhibit the production of proinflammatory factors, and promote tissue repair and regeneration. However, single-cell RNA sequencing revealed that microglia present a variety of intermediate phenotypes and cell clusters with different transcript levels. Their functional responses are highly specific and dynamic, and the boundaries between M1/M2 phenotypes are blurred and can be transformed into each other (Li et al., 2022c; Tansley et al., 2022; Yao et al., 2022).

After SCI, microglia initially exhibit a transient M2 phenotype that promotes repair and anti-inflammatory effects. In the context of sustained disease microenvironment stimuli, these microglia respond quickly, their numbers increase, and they transform from the M2 phenotype to the more aggressive M1 phenotype (Fang et al., 2023; Wei et al., 2023b). During the progression from the subacute phase to the chronic phase, M1 microglia dominate the inflammatory response, which not only exacerbates tissue damage but also significantly hinders subsequent repair processes. Extracellular ATP is released in large quantities as a damage-related molecular pattern signaling molecule, activating the purinergic P2 signaling cascade in microglia (Elliott et al., 2009). Receptors such as P2X4, P2X7, P2Y6, P2Y12, and P2Y13, which are expressed by microglia, are involved in both the inflammatory response and the repair process (**Figure 5**). On one hand, P2YR-mediated signaling promotes microglial phagocytosis and the inflammatory response. If the binding of this receptor to ATP is inhibited, the phagocytosis and elimination ability of microglia will be weakened. On the other hand, under certain conditions, P2X7 receptor-mediated high-concentration ATP pretreatment can reduce neuronal damage (Masuch et al., 2016). For example, it can inhibit the activation of microglia and reduce the expression of proinflammatory factors such as TNFα, IL-6, and IL-12. These findings highlight the key and complex role of purine signaling in the regulation of SCI inflammation and provide potential targets for therapeutic intervention.

*Microglia and scar formation after spinal cord injury*

Microglia provide a material basis for the construction of scar tissue by secreting extracellular matrix components such as CSPG. Although CSPG limits axonal regeneration to a certain extent, it helps isolate inflammation, blocks the invasion of macrophages, and prevents damage diffusion (Jakovčevski et al., 2021; Zheng et al., 2023). Different phenotypes of microglia play different roles in this process. Specifically, the M1 type tends to promote scar formation and aggravate axonal regeneration. In contrast, the M2 type may inhibit excessive scar hyperplasia and facilitate tissue repair (Mishra et al., 2021). In the repair of SCI in neonatal mice, microglia-mediated scar-free healing and inflammatory regulation lead to the regeneration of many axons. This finding is in stark contrast to that in adult mice, suggesting that the developmental stage of microglia affects the mechanism of scar formation. Moreover, the Plexin-B2 signaling pathway plays a key role in wound containment and compression of microglia and macrophages (Zhou et al., 2020). In the early stage of injury, Plexin-B2 is highly expressed in activated cells, limiting cell proliferation and reducing tissue damage. The above results show that microglia have diverse functions in scar formation, so an in-depth study of their mechanism is highly important for optimizing SCI treatment and promoting axon regeneration.

*Effects of microglia on blood vessels after spinal cord injury*

Microglia interact closely with vascular endothelial cells, thus profoundly affecting BSCB function and angiogenesis. SCI-induced hypoxia, ischemia, and cytokine storms promote microglial activation and the release of reactive oxygen species, NO, iNOS, IL-6, and matrix metalloproteinases (Smith et al., 2022). ROS and NO damage endothelial cells, destroy tight junction proteins, increase BSCB permeability, and cause peripheral white blood cells to infiltrate the CNS parenchyma (Yao et al., 2022; Jaffer et al., 2023). Moreover, IL-6 activates the STAT3 signaling pathway in endothelial cells, downregulates the expression of tight junction proteins, cooperates with matrix metalloproteinases to degrade the extracellular matrix, aggravates BSCB damage, and expands the scope of damage. However, microglia also have the potential to protect blood vessels. In CNS vascular injury, local microglia stabilize blood vessels in a purinergic receptor-dependent manner, forming tubular structures to reduce leukocyte infiltration (Mastorakos et al., 2021; Zhang et al., 2021). These results highlight the duality and complexity of the role of microglia in blood vessels, thus providing a new perspective for the treatment of SCI-related vascular repair.

*Interaction between microglia and other cells after spinal cord injury*

There is a complex two-way regulatory relationship between microglia and neurons after SCI (Lukacova et al., 2021). On one hand, the activation and proliferation of microglia promote the release of proinflammatory factors and reactive oxygen species, leading to axonal degeneration and demyelination and causing direct damage to neurons (Huang et al., 2023; Zhang et al., 2024c). On the other hand, neuronal damage or death can release signaling molecules to activate microglia and form a vicious cycle. However, under certain conditions, microglia can secrete neuroprotective factors such as BDNF (Jia et al., 2023). These factors promote neuronal regeneration and repair and enhance neural signal transmission through physical contact and purinergic signal transduction (Pottorf et al., 2022). The interaction between microglia and neurons has become a key link in the treatment of SCI. For example, bumetanide can regulate the characteristics of microglia and improve the function of neurons, providing a direction for drug development. In addition, the interaction between microglia and astrocytes is also an important part of the pathological process of SCI. After SCI, microglia secrete factors such as IL-1α, TNF-α, and C1q, induce astrocytes to transform into the neurotoxic A1 phenotype, promote their proliferation and activation, and form glial scars (Kwon and Koh, 2020; Zhang et al., 2024c). Moreover, astrocytes secrete proinflammatory substances such as granulocyte‒macrophage colony‒stimulating factor, which further increase the activation of microglia and the production of TNF-α and aggravate inflammatory injury (Kano et al., 2019). However, in some inflammatory situations, astrocytes can secrete TGF-β and galectin-1 to inhibit microglial activation and have a positive effect (Kabba et al., 2018). Therefore, an in-depth analysis of this interaction mechanism is highly important for regulating the SCI inflammatory response and promoting repairs.

#### Effects of exercise training on the repair and regeneration of microglia after spinal cord injury

*Exercise training regulates microglial activation*

In the study of the effect of exercise training on the activation of microglia after nerve injury, the selection of different exercise conditions and animal models reveals a complex and changeable effect model, which provides a rich perspective for further understanding this biological phenomenon (Arbat-Plana et al., 2019). A study of male rats revealed that both high-frequency and low-frequency treadmill exercise training significantly inhibited the activation of microglia and astrocytes in the spinal dorsal horn. Specifically, the immunoreactivity of microglia for Iba1 was significantly reduced (Sumizono et al., 2018). These findings highlight the positive role of exercise training in regulating glial cell activity and reducing neuroinflammatory responses. However, when we turned our attention to the thoracic spinal cord contusion model of adult female SD rats, the situation was different. Despite the use of treadmill exercise training (TMT), no increase in the number of injury-induced microglia was observed, and the synaptic remodeling mediated by microglia was not significantly regulated (Shin et al., 2014). Furthermore, a study by Manthou et al. (2017) highlighted the criticality of the treatment window period, providing new insights into the optimization of treatment initiation time and frequency. In particular, whole-body vibration (WBV) is initiated during the subacute phase of SCI, which can more effectively inhibit the excessive activation of microglia and promote the recovery of neurological function. In terms of training frequency, the arrangement of 3–5 times a week has been proven to be a better choice by many studies (Stagg et al., 2011; Sumizono et al., 2018). This frequency can not only provide sufficient physiological stimulation to promote neural plasticity but also prevent the aggravation of fatigue and stress responses caused by overtraining in animals (and even human patients).

Furthermore, a study in mouse models also revealed the diverse effects of exercise training on microglial activation (Mifflinet al., 2017). Using an EAE model in C57BL/6J mice, researchers reported that voluntary wheel running cannot regulate spinal Iba-1 levels due to EAE. However, it is worth noting that in a sex comparison, the activation level of microglia in male mice was significantly greater than that in female mice (Mifflinet al., 2017). In addition, a study based on a complete spinal cord transection model in female CD1 mice combined with lumbar muscle injection of complete Freund’s adjuvant (CFA) revealed that although step training improved motor function, it did not effectively regulate the increased expression of microglia (Jeffrey-Gauthier et al., 2021). Moreover, short-term treadmill running in CD1 male mice had a positive effect on reducing the number of microglia and astrocytes in the L4/L5 segment of the spinal cord and reducing abnormal pain. However, long-term running is not conducive to recovery and can even lead to increased expression of astrocytes (Cobianchi et al., 2010; Bai et al., 2022). On the basis of the differences in the results of the above studies, we speculate that exercise training may not be able to completely reverse the trend of glial cell activation in some cases of extreme nerve injury.

In summary, the effect of exercise training on microglial activation is a complex and multidimensional process that is restricted by many factors, such as sex, animal model, injury type, training method and duration. Therefore, future research needs to explore the interaction mechanism between these factors more deeply and develop more accurate and effective exercise intervention strategies to achieve the best therapeutic effect.

*Exercise training promotes axon regeneration*

Exercise training has shown significant potential in promoting nerve repair and functional recovery after SCI. The mechanism is complex and multidimensional, especially in terms of the recovery of synaptic density, synaptic plasticity and microenvironment regulation (Jo et al., 2021; Skiadopoulos et al., 2023). These effects not only provide favorable conditions for axon regeneration and neural network reconstruction but also profoundly affect the phenotypic switching of microglia and their associated biological processes (Gouveia et al., 2022; Yu et al., 2024).

In a rat model of severe compressive SCI (Wirth et al., 2013), exercise training effectively enhanced body weight support and promoted the restoration of neural connectivity by increasing the synaptic density in the lumbar segment of the spinal cord. This process may involve fine-tuning of synaptic plasticity, creating a more favorable microenvironment for axonal regeneration and neural repair. In a moderate contusion SCI model (Wang et al., 2015), exercise training demonstrated positive effects by significantly improving the motor function of rats. Additionally, it increased the total dendritic length, BDNF expression, and synaptic density of lumbar motor neurons. These changes play crucial roles in promoting axon growth and the reconstruction of nerve connections. Moreover, exercise training can increase the activity of matrix metalloproteinases (MMPs), promoting the degradation and remodeling of collagen and fibronectin. This process increases the flexibility and permeability of the extracellular matrix, facilitating the migration of microglia and supporting axon regeneration (Jakeman et al., 2011; Schreiber et al., 2014; Ying et al., 2020). Moreover, exercise training can promote the synthesis of some extracellular matrix proteins, maintain their normal structure and function, and create an ideal microenvironment for nerve regeneration.

Notably, microglia, as key immune cells in the CNS, play important roles in the modulation of neuroinflammation and the creation of a neurorestorative microenvironment due to their phenotypic transition (especially from M1 to M2) (Zhou et al., 2024). After nerve injury in young rats, microglia in the spinal cord initially exhibit M2-type polarization, which is beneficial for neuroprotection. However, as they enter puberty, these cells change to the M1 type, accompanied by the emergence of neuropathic pain. Interestingly, exercise training can promote the polarization of microglia from the M1 to the M2 phenotype. This shift is accompanied by an increase in the expression of the anti-inflammatory factor IL-10 and a decrease in the expression of the proinflammatory factor TNF-α, which effectively reduces neuropathic pain (Gong et al., 2017; Bai et al., 2022). Although direct evidence remains to be obtained, the neural repair microenvironment created by M2 microglia undoubtedly plays an indirect role in promoting axonal regeneration.

In a study of female SD rats, different intensities of weight support treadmill training had different effects on recovery (de Leon et al., 2011). In particular, the recovery of motor function in rats after high-intensity training (1000 steps per day) was more significant, and the level of synaptophysin immunoreactivity around motor neurons was close to normal. In contrast, low-intensity training (100 steps per day), although less effective, also increased the immunoreactivity of BDNF. These results suggest that the optimal combination of training intensity and duration should be fully considered to achieve the best recovery effect.

In recent years, the rise of brain–computer interface (BCI) technology has provided new perspectives and possibilities for the rehabilitation of SCI patients (Massoto et al., 2020). By directly linking cortical activity to muscle electrical stimulation, BCIs can bypass the lesion area and restore the function of paralyzed limbs (Hu et al., 2023). Studies have shown that the combination of BCIs and exercise training can significantly improve the motor ability and functional recovery after SCI patients. This finding further expands the boundaries of SCI rehabilitation strategies.

*Exercise training regulates the phagocytosis of microglia*

After SCI, the complement system is often activated, and complement components such as C1q and C3 are used to label damaged tissues and cell debris, which in turn triggers the phagocytosis and clearance mechanism of microglia (Stevens et al., 2007; Cong et al., 2020; Han et al., 2024a). However, excessive activation of the complement system may trigger excessive phagocytosis of normal nerve tissue by microglia, thereby exacerbating nerve damage. Exercise training effectively reduces the level of complement by negatively regulating the complement pathway, thereby alleviating the excessive phagocytosis of normal tissues by microglia. Specifically, exercise training may inhibit the activity of key enzymes (such as C1 esterase) in the complement activation pathway by regulating the expression of complement-related genes, thereby reducing the production of complement components (such as C1q and C3) (Wang et al., 2023a; Yang et al., 2023). In addition, exercise may promote the expression of complement regulatory proteins such as CD55 and CD59, which inhibit complement activation and the formation of membrane attack complexes, helping to protect nerve cells and synapses from complement-mediated damage (van Beek et al., 2005; Wang et al., 2010; Li et al., 2013).

In addition to regulating the complement system, exercise training may also finely regulate phagocytic function by affecting the expression of phagocytic receptors on the surface of microglia (Yao et al., 2024). Exercise may increase the expression of some beneficial phagocytic receptors on the surface of microglia, promote their effective removal of cell debris and toxic substances at the injured site, and prevent further damage to surrounding nerve cells by these harmful substances (Zhang et al., 2024a, c). Moreover, exercise training may reduce the expression of receptors associated with excessive phagocytosis, thereby preventing the misphagocytosis of normal nerve tissue by microglia and protecting the integrity of nerve structure and function (Wei et al., 2023b). After SCI, complement components may bind to adjacent weaker synapses and engulf them through activated microglia, leading to synaptic loss and neurological dysfunction. Exercise training reduces excessive synapse phagocytosis by microglia by inhibiting complement activation, thereby helping to protect neural circuits (Yao et al., 2024).

In summary, exercise training regulates the expression of phagocytic receptors on the surface of the complement system and microglia through multiple pathways. Moreover, exercise training can finely regulate the phagocytic function of microglia, promote the repair and regeneration process after SCI, and help to restore nerve function. These findings provide new perspectives and strategies for the rehabilitation of SCI patients.

## Clinical Translation

In recent years, exercise training has made remarkable progress in promoting the clinical translation of nerve repair after SCI (Wagner et al., 2018). The depth and breadth of research in this field have been continuously expanded, covering the development of new drugs, the application of innovative instruments, extensive clinical trial exploration and in-depth basic scientific research. These efforts have not only been strongly supported by the Food and Drug Administration (FDA) (https://www.fda.gov/), the ClinicalTrials.gov (https://clinicaltrials.gov/), the Chinese Clinical Trial Registry (https://www.chictr.org.cn/index.html) and the National Institutes of Health (NIH) (https://www.nih.gov/) but also promoted interdisciplinary and interdisciplinary cooperation.

In the research and development of new drugs and devices for SCI, although no specific drugs have directly targeted nerve repair and regeneration in the past three years, foundational research on potential therapeutic agents, such as the TTK21 small molecule (Singh et al., 2024), has generated new avenues for future drug treatments. Additionally, a series of innovative devices approved by the FDA, including the Atalante X exoskeleton system, the ARC-EX spinal cord stimulation system, and the NeuRx DPS® diaphragm pacemaker (https://www.fda.gov/), have effectively advanced nerve repair and functional rehabilitation in SCI patients through physical assistance and nerve stimulation, providing a robust practical foundation for clinical translation. These achievements not only highlight the impact of technological innovation but also highlight the critical role of interdisciplinary collaboration in advancing medical progress.

In the field of clinical trials, a number of studies registered by the North American Clinical Trial Registry and the Chinese Clinical Trial Registry have explored the feasibility, safety and effectiveness of specific forms of exercise, such as high-intensity interval training, in the rehabilitation of patients with SCI (https://www.chictr.org.cn/index.html). These studies not only combine modern scientific and technological means such as transcutaneous electrical stimulation of the spinal cord, BCI technology, and magnetic stimulation but also aim to develop more effective and personalized rehabilitation programs (https://clinicaltrials.gov/). The development of the above clinical trials provides not only a new dawn of rehabilitation for patients but also valuable clinical data and theoretical support for basic research in the field of neurorehabilitation.

In the field of basic research, NIH-supported projects related to SCI nerve repair have yielded remarkable results. Animal experiments have provided in-depth insights, with significant findings emerging from models such as zebrafish (Saraswathy et al., 2024). In a study focused on axonal regeneration (Anderson et al., 2018), key factors, including gene manipulation to promote the early growth recovery of neurons, the construction of axonal pathways through damaged tissue, and the guidance of axon growth beyond the injury site, were identified. Research on regeneration in adult nerve cells after SCI has revealed mechanisms related to energy deficiency in injured cells and the role of the protein syntaphilin in hindering axonal regeneration and the mitochondrial energy supply. By utilizing specific mouse models and conducting creatine intervention experiments (Han et al., 2020), the crucial role of energy in this process has been preliminarily confirmed, indicating pathways for the subsequent exploration of high-efficiency energy compounds in the CNS and facilitating the transition to clinical treatment programs. Additionally, 3D printing technology has been employed to create custom hydrogel implants that simulate spinal cord tissue. In rat experiments, implants loaded with embryonic neural stem cells facilitated new nerve cell connections, vascular regeneration at the SCI site, and recovery of hindlimb function. This research has the potential to produce implants for complex human SCI cases, with plans for further refinement and future human clinical trials, providing innovative therapeutic options for SCI (Koffler et al., 2019). Moreover, single-cell gene analysis of spinal cord neurons (Osseward et al., 2021) and astrocyte-associated therapies (Dutta et al., 2018) have accurately identified disease-related neurons and clarified the mechanisms of intercellular interactions, laying a solid foundation for accurate and personalized medicine.

In current clinical rehabilitation practices, exercise therapy has emerged as a cornerstone strategy in SCI rehabilitation, with its precision and individualization increasingly driven by robust research and evidence. For patients with incomplete SCI, early intervention (within 60 days postinjury) with high-intensity gait training capitalizes on the critical window of neuroplasticity, significantly improving walking capacity (Fahey et al., 2022; Henry et al., 2024). Additionally, robot-assisted gait training (RAGT) has demonstrated exceptional efficacy during the subacute phase and in interventions beyond two months postinjury, offering robust technical support for lower limb functional reconstruction (Park et al., 2024b). Moreover, endurance training enriches rehabilitation options by promoting neural regeneration and bladder functional recovery, adding new dimensions to exercise-based therapeutic protocols (Kiss Bimbova et al., 2023). However, the application of high-intensity training necessitates a strict balance between safety and efficacy. Dynamic assessment of patients’ physical conditions and timely adjustment of training intensity are crucial. For example, standardized evaluation tools such as the 6-minute walk test are recommended to monitor rehabilitation progress and ensure scientific rigor in training programs (Gaspar et al., 2019). These findings collectively underscore the importance of personalized therapy in SCI rehabilitation. When exercise prescriptions are designed, it is essential to integrate injury severity, age, and functional status to establish stepped therapeutic protocols, thereby achieving precision in rehabilitation goals (Bertels et al., 2023).

Clinical research on the ability of exercise therapy to promote neural repair remains intricate and challenging but continues to achieve new breakthroughs. The primary research focus lies in elucidating the temporal window of neural repair and its underlying molecular mechanisms, with particular emphasis on identifying biomarkers for personalized intervention strategies. Furthermore, Sandrow-Feinberg and Houlé (2015) aimed to investigate the regulatory mechanisms of key signaling pathways, such as the mTOR pathway, during endurance training, thereby establishing a theoretical foundation for precision medicine in neural rehabilitation.

Technological innovation is pivotal, particularly in developing intelligent rehabilitation systems. In terms of technological innovation, the development of intelligent rehabilitation systems is of paramount importance. This involves the integration of RAGT with virtual reality technology to create immersive training environments, as well as the application of AI algorithms for real-time optimization of motion parameters to increase training efficacy (Park et al., 2024b). Moreover, the exploration of combined therapeutic modalities is underway, focusing on the synergistic application of transcranial magnetic stimulation and robotic-assisted training, along with the development of home-based rehabilitation programs supported by remote monitoring systems. These initiatives aim to deliver more accessible and efficient rehabilitation services to patients (Duan et al., 2021). In addition, establishing multidimensional evaluation models encompassing functional recovery, quality of life, and social participation is crucial to drive the evolution of exercise therapy from isolated functional improvement toward comprehensive rehabilitation paradigms.

## Limitations

Although this review provides a comprehensive and in-depth discussion of how exercise training promotes nerve cell repair and regeneration after SCI, several limitations remain.

In terms of research comprehensiveness, while this review addresses several major neuronal cell types, such as neurons, astrocytes, and microglia, it does not thoroughly explore the changes in and mechanisms of other cell types that may be involved in the repair process under the influence of exercise training. For example, a recent study demonstrated that downhill exercise training promotes oligodendrocyte generation in the cervical dorsal and lateral white matter of spinal cord-injured mice, enhances interactions between axons and oligodendrocytes, and restores paranodal structures in the dorsal column of sensory ganglia (Faw et al., 2021). Additionally, exercise training has been shown to stimulate the proliferation and activation of ependymal cells. For example, forced running wheel training can significantly increase BrdU immunoreactivity in ependymal progenitor cells lining the central canal of adult rat spinal cords, indicating exercise-mediated endogenous ependymal cell responses (Maugeri et al., 2024). Although existing research has highlighted the positive effects of exercise training on oligodendrocytes and ependymal cells after SCI, a comprehensive exploration or synthesis of these mechanisms has not been performed. This gap may hinder a holistic understanding of the processes related to injured spinal cord repair. This oversight may limit the overall understanding of the mechanisms involved in injured spinal cord repair. Furthermore, although various mechanisms by which exercise training impacts different types of nerve cells have been investigated, the intricate signal transduction and molecular regulatory networks involved in the pathological processes of SCI and the cellular responses to exercise intervention remain unclear. Specifically, the cross-talk mechanisms between different cell types, the details of how noncoding RNAs synergistically regulate transcription factors and protein signaling molecules, and the dynamic evolution of epigenetic modifications during the long-term repair process have not been explored in detail. The incomplete understanding of these mechanisms hinders a comprehensive analysis of how exercise promotes nerve repair and the development of targeted therapeutic strategies. With respect to molecular mechanisms, while multiple signaling pathways and molecules are mentioned, some mechanisms remain in the preliminary stages of research. For example, the specific transcriptional regulatory networks through which exercise training regulates the expression of nerve growth factors have not been fully elucidated, and the synergistic or antagonistic relationships between transcription factors, as well as their precise regulation by exercise training, still require further investigation.

In terms of clinical transformation, while some potential directions for new drug development and approved interventions have been identified, the evaluation of their specific effects in practical clinical applications is insufficiently detailed. In the context of clinical trials, the unique characteristics of SCI patients, such as genetic polymorphisms, differences in epigenetic modifications, neuroendocrine regulation, and psychological resilience, have not been adequately considered in relation to their responses to exercise training. These individual factors can affect cellular sensitivity to exercise stimulation, signal transduction efficiency, and overall repair and regeneration capabilities. As a result, existing group-based exercise programs may not adequately address the specific needs of each patient, ultimately impacting the optimization of treatment outcomes.

## Conclusion and Prospects

This review comprehensively examines the progress of research on the ability of exercise training to promote the repair and regeneration of nerve cells after SCI. In recent years, the field of neural repair for SCI has made remarkable progress, with numerous researchers making outstanding contributions. For example, the integration of robot-assisted rehabilitation and DBSLH therapy has been demonstrated to significantly increase the projection number of vGi neurons to the lumbar spinal cord, thereby effectively improving patients’ walking ability (Cho et al., 2024). However, despite these advancements, the field still faces multiple challenges. Currently, the precise molecular mechanisms by which motor training promotes neural repair remain incompletely understood, particularly with respect to the interactions among different cell types, the cooperative regulation of noncoding RNAs and transcription factors, and the dynamic changes in epigenetic modifications. Additionally, significant challenges persist in clinical translation. Current research is predominantly limited by small-scale trials and a lack of systematic long-term follow-up data, which hinders the direct application of research findings to clinical practice.

This review offers an in-depth exploration of how exercise training promotes neural cell repair and regeneration after SCI from various perspectives. First, this work systematically analyzes the pathological alterations in neural cells after SCI, comprehensively revealing the complexity of SCI, which includes the impairment of intrinsic regenerative capacity in neurons, disruption of microenvironment homeostasis, and abnormal activation of glial cells, among other critical pathological features. Second, this review thoroughly examines the specific mechanisms through which exercise training impacts neurons, astrocytes, and microglia, elucidating its multifaceted role in facilitating neural repair. Furthermore, by incorporating recent research advancements such as epigenetic regulation and emerging fields such as RAGT, this review provides new insights into the therapeutic effects of exercise training.

The significance of this review lies in establishing new theoretical foundations and practical guidance for SCI rehabilitation. By systematically elucidating the molecular mechanisms through which exercise training promotes neural cell repair and regeneration, this review fully demonstrates the enormous potential of exercise training in improving neurological functional recovery, thereby offering new research directions and treatment paradigms for the rehabilitation of clinical SCI patients. Moreover, this review synthesizes current progress and challenges in the clinical translation of exercise-based interventions, providing valuable references for future research directions.

Future research should further elucidate the molecular mechanisms underlying exercise training-induced neural repair, particularly focusing on interactions among distinct cell types and epigenetic regulatory mechanisms. Large-scale, multicenter clinical trials with long-term follow-up, which employ clearly defined patient inclusion criteria and standardized exercise training interventions, are recommended to elucidate the specific effects of exercise training on different SCI patient subtypes. Additionally, interdisciplinary collaborations should be strengthened to leverage big data analytics and AI algorithms in exploring correlations between clinical data and exercise training parameters, ultimately establishing personalized rehabilitation models. Finally, the development of intelligent rehabilitation systems incorporating cutting-edge technologies, including virtual reality and robotic-assisted training, is recommended to increase the efficacy and therapeutic outcomes of rehabilitation interventions, thereby offering more comprehensive and tailored rehabilitation support for individuals with SCI.

## Additional files:

***Additional Table 1:*** *Effects of exercise training on the intrinsic growth capacity of neurons.*

***Additional Table 2:*** *Effects of exercise training on the microenvironment of neuron growth.*

***Additional Table 3:*** *Exercise training mediates the repair effect of astrocytes on SCI.*

## Data Availability

*All relevant data are within the paper and its Additional files*.
